# Characteristics of allelic gene expression in human brain cells from single-cell RNA-seq data analysis

**DOI:** 10.1186/s12864-017-4261-x

**Published:** 2017-11-10

**Authors:** Dejian Zhao, Mingyan Lin, Erika Pedrosa, Herbert M. Lachman, Deyou Zheng

**Affiliations:** 1 Department of Neurology, Albert Einstein College of Medicine, 1300 Morris Park Ave., Bronx, NY USA; 2 Department of Genetics, Albert Einstein College of Medicine, 1300 Morris Park Ave., Bronx, NY USA; 3 Department of Psychiatry and Behavioral Sciences, Albert Einstein College of Medicine, 1300 Morris Park Ave., Bronx, NY USA; 4 Department of Neuroscience, Albert Einstein College of Medicine, 1300 Morris Park Ave., Bronx, NY USA; 5 Department of Medicine, Albert Einstein College of Medicine, 1300 Morris Park Ave., Bronx, NY USA; 6 Present address: Department of Neuroscience, School of Basic Medical Science, Nanjing Medical University, Nanjing, Jiangsu 21166 China

**Keywords:** Allelic gene expression, Single-cell RNA-seq, Human brain

## Abstract

**Background:**

Monoallelic expression of autosomal genes has been implicated in human psychiatric disorders. However, there is a paucity of allelic expression studies in human brain cells at the single cell and genome wide levels.

**Results:**

In this report, we reanalyzed a previously published single-cell RNA-seq dataset from several postmortem human brains and observed pervasive monoallelic expression in individual cells, largely in a random manner. Examining single nucleotide variants with a predicted functional disruption, we found that the “damaged” alleles were overall expressed in fewer brain cells than their counterparts, and at a lower level in cells where their expression was detected. We also identified many brain cell type-specific monoallelically expressed genes. Interestingly, many of these cell type-specific monoallelically expressed genes were enriched for functions important for those brain cell types. In addition, function analysis showed that genes displaying monoallelic expression and correlated expression across neuronal cells from different individual brains were implicated in the regulation of synaptic function.

**Conclusions:**

Our findings suggest that monoallelic gene expression is prevalent in human brain cells, which may play a role in generating cellular identity and neuronal diversity and thus increasing the complexity and diversity of brain cell functions.

**Electronic supplementary material:**

The online version of this article (10.1186/s12864-017-4261-x) contains supplementary material, which is available to authorized users.

## Background

In diploid eukaryotic organisms, it is generally thought that the maternal and paternal copies of individual genes are expressed simultaneously at comparable levels. However, there are exceptions where only one of the two alleles is expressed; chromosome X-linked genes and imprinted genes are the best-known examples [1]. In addition, monoallelic expression of autosomal genes has also been observed in several large gene families that are active in the nervous or immune systems, such as the olfactory receptor gene family [2, 3], protocadherins [4, 5], interleukins and immunoglobulins [6]. There, monoallelic expression is functionally essential for generating cellular identity and diversity [2, 7, 8]. Moreover, recent transcriptome-wide analyses have showed that monoallelic expression is much more widespread than previously appreciated [1, 9–13]. The extent of monoallelic expression, however, remains unclear and is subject to debate, as the experimental technology and the operational definition of monoallelic expression vary from study to study. Arguably, many of the previous studies actually investigated allele-biased expression rather than monoallelic expression, including our published work [14], as pointed out by recent reports [15, 16].

When allelic expression occurs in humans, it may be uniformly biased to the same allele in one tissue or organ, or throughout the body, or the two alleles may be expressed randomly. Conceivably, random monoallelic expression can contribute to developmental disorders when this occurs in a gene containing heterozygous loss-of-function mutations. A known example is the X-linked *MECP2* gene. It is mutated in Rett Syndrome and approximately half of the cells in a female patient would be expected to express the mutated copy, leading to disrupted cellular functions [17, 18]. Likewise, autosomal genes undergoing monoallelic expression may also be implicated in human disorders. For example, the *AGC1* gene, which leads to a severe developmental abnormality with loss of function mutations, has been shown to be expressed monoallelically in a random manner in mice [19]. Monoallelic expression of *APP* and *SNCA* may also be involved in the risk of Alzheimer and Parkinson diseases, respectively [9, 20]. The functional impacts of monoallelic gene expression, however, remain largely unclear.

To study monoallelic expression and its potential role in human brain disorders, both in vitro cell cultures and post-mortem brain samples have been employed. Our previous study identified many allele-biased expressed genes in induced pluripotent stem cells (iPSCs) and differentiated neurons, some of which are implicated in schizophrenia and autism [14]. The finding was supported by other investigators [21]. Two recent studies found that the establishment of monoallelic gene expression during embryonic stem cell (ESC) differentiation was stably maintained over multiple cell divisions [20, 22]. When ESC cells were differentiated into neural progenitor cells (NPCs), however, the monoallelic expression pattern can be reset [22]. On the other hand, when NPCs were further differentiated into other neural cells such as astrocytes, the allelic expression patterns seemed to be preserved [20]. This discovery may explain why some disease-related mutations show variable penetrance and supports the hypothesis that monoallelic expression may be a reason for discordance for monozygotic twins in human diseases such as schizophrenia and Parkinson Disease, as the mutated gene copies may not be expressed in all individuals or among all cells [14]. Consistent with this idea, several genes important for neurodevelopment and implicated in neurological disorders, such as ASD, intellectual disability, and developmental delay have been shown to exhibit monoallelic expression, such as *AUTS2* (autism susceptibility candidate 2) in lymphoblastoid cell lines [23] and human neurons [14]. Studies have also observed that genes encoding the GABAA-receptor subunits *GABRB3*, *GABRA5* and *GABRG3* showed allele-biased expression in the frontal cortex of ASD individuals but not in controls [24], while *SLC1A3* and *NHP2L1* displayed allele-biased expression in selected brain regions [25]. Most recently, Huang et al. reported that allelic effects were developmental stage and cell type specific, and they found that the allelic expression of genes, including risk genes for mental disorders, could give rise to mosaics of monoallelic and biallelic expression in macaque and human brain cells [26]. Despite these important findings, little is known about monoallelic expression in individual human brain cells.

Furthermore, as a brain is composed of various cell types, it is difficult to interpret transcriptome data derived from whole brains for studying allelic expression and understanding cellular functions. The development of single-cell RNA-seq (scRNA-seq), however, has made it feasible to study gene expression of all brain cells at the same time and resolve expression profiles down to individual cells. The first scRNA-seq of brain cells was conducted on fetal cortical tissue samples, in which the authors revealed the heterogeneity of gene expression in individual cells and discovered that the Notch signaling pathway is activated in human radial glia [27]. Another scRNA-seq of brain cells was conducted on both fetal (*n* = 4) and adult cortical samples (*n* = 8) [28]. The authors used two complementary approaches to classify the adult brain cells into five major cell types: astrocytes, microglia, neurons, oligodendrocytes, and oligodendrocyte precursor cells (OPC) [28]. An independent study separated neural progenitors in human fetal cortex by fluorescence-activated cell sorting, analyzed the sorted progenitors using RNA-seq, and found that both neurogenin targets and long noncoding RNAs were enriched in human outer radial glia [29]. Finally, an RNA-seq study using human prenatal brain tissues echoed the importance of lncRNAs in human neocortex development, as it demonstrated that LOC646329, one of the most radial glia-enriched lncRNAs, regulated cell proliferation [30]. In short, these scRNA-seq studies have uncovered brain cell heterogeneity, mapped gene signatures for different cell types, provided invaluable resources for investigating gene expression of brain cells, and highlighted the importance of studying gene expression at the single cell level.

Here, we studied the scRNA-seq datasets from several adult human brains [28] and re-analyzed them for allele-biased gene expression. We found that monoallelic gene expression occurred widely in brain cells and the monoallelic genes tended to be cell type-specific. When compared to co-expressed gene modules, monoallelic genes were enriched in the neuron module, indicating that these genes may be important for neuronal specification and functions.

## Results

### Method to call single nucleotide polymorphism (SNP) from scRNA-seq datasets

**Fig. 1:**
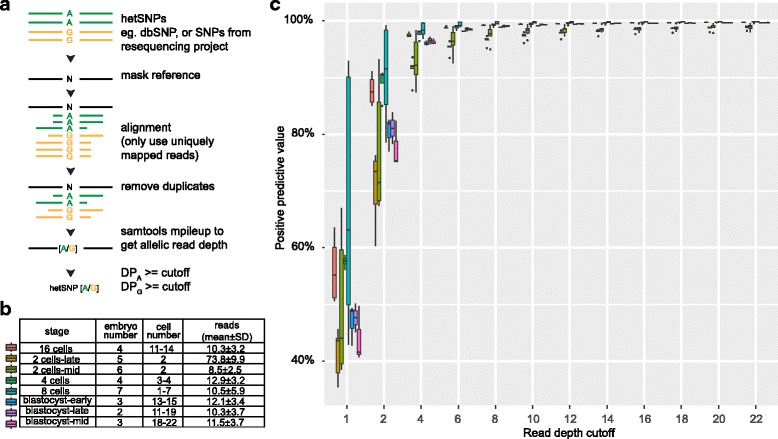
Accuracy of hetSNP calling from scRNA-seq data. **a** Method to compute allelic read depths at candidate SNP sites. **b** Summary of the mouse scRNA-seq dataset from 34 embryos at different developmental stages; each stage had multiple embryos and a different number of single cells (the minimal and maximal are provided) were analyzed for each embryo. Uniquely mapped reads in cells are presented as mean ± SD (in millions). **c** The percentages of hetSNPs (y-axis) called from mouse F1 embryos that were consistent with genotyping data. Boxplots are used to summarize data from individual embryos of the same developmental stages (see color legend in **b**). The x-axis depicts read depth cutoffs used in calling hetSNPs

Heterozygous DNA markers are required for allelic expression analysis. These are usually derived from SNP genotyping data [14, 20, 22]. In this study, we re-analyzed previously published single cell RNA-seq data, for which genotype information is not available. We reasoned that a subset of heterozygous SNPs (hetSNPs) in an individual subject could be discovered directly by pooling scRNA-seq data from all cells, since the two alleles of a gene could be expressed in different single cells [12, 31, 32]. By largely following the SNP calling method applied to bulk RNA-seq data [33, 34], we developed a hetSNP calling pipeline using pooled scRNA-seq data and information from the dbSNP database (see Methods; Fig. 1a). To test our method, we first applied it to a mouse embryonic scRNA-seq dataset with available genotying information. In the dataset, RNAs from 269 single cells from multiple F1 embryos at different developmental stages - from zygote to late blastocyst - were sequenced, with cell numbers ranging from 1 to 27 [12]. We excluded the scRNA-seq data of four zygotes and four early 2-cell embryos (Fig. 1b) because we found very few hetSNPs from those data (on average 22 for zygotes and 35 for early 2-cell embryos), which is consistent with the fact that the paternal genome is not fully activated at these two stages and thus the maternal alleles were the predominant alleles for most genes [12]. We masked the 58,817,922 SNP sites (dbSNP version 142) in the mouse reference genome, among which 17,491,332 sites are known heterozygous SNPs (hetSNPs) between the two mouse strains, CAST/Ei and C57BL/6, used to generate the F1 embryos. We called SNPs for 34 embryos after merging scRNA-seq data from different cells of the same embryo (Additional file 1: Figure S1a). Comparing the hetSNPs called from the scRNA-seq data in each embryo with the list of known hetSNPs derived from the mouse genome project (Additional file 1: Figure S1b), we calculated the positive predictive values (Fig. 1c). For all the 34 tested embryos, regardless of different cell numbers and depths in the scRNA-seq data, the positive predictive values increased dramatically from 53.09% to 97.20% on average when the read depth cutoff was increased from 1 to 6, after which the positive predictive values reached a plateau phase. To be highly conservative while keeping a reasonable number of hetSNPs for analysis, we decided to use the cutoff of 20 for each of the two alleles in pooled RNA-seq data, yielding a positive predictive value of 99.46% on average (Fig. 1c). Although the positive predictive values were high, the true positive rate, as expected, was small (Additional file 1: Figure S1c) due to various reasons. For example, most hetSNPs were located at intergenic or regulatory regions and could not be detected in RNA-seq due to the lack of expression. As an alternative option, we tried to call SNPs directly from pooled RNA-seq data using the GATK at the genome-wide level [35, 36]. However, this resulted in much fewer hetSNPs (min 100 SNPs, max 3037 SNPs; ~7% of hetSNPs identified above), of which 77% were present in the dbSNP. Moreover, we compared the allelic ratios of the SNPs from the GATK pipeline and those from our method, and found the allelic ratios of most SNPs missed by GATK were deviated from 1:1 (Additional file 1: Figure S1d). Considering this finding, we have decided not to pursue this option for calling variants.

### Identification of hetSNPs in individual human brains

After testing the SNP calling method on mouse scRNA-seq data, we applied it to a human scRNA-seq dataset that contained 466 cells from eight adult and four fetal brains [28] (Additional file 2: Table S1). The original study provided a clear overview of cellular heterogeneity and complexity of the adult and fetal human cortical regions at the single cell transcriptome level [28]. Here, we re-analyzed the dataset to study the pattern of allelic expression across cell types in human adult brain cells, using the cell type classification provided by the original authors. Since the fetal brain cells were not mapped to specific cell types, they were excluded from current analysis. We also excluded the two adult samples with only four and five cells. In the end, we analyzed 323 single cells from six adults (adult21, adult37, adult47, adult50, adult54, and adult63B) (Additional file 2: Table S1). We first called SNPs in each adult using the method described above (Fig. 1a; Additional file 1: Figure S2). Again, we tried a series of read depth cutoffs. When the read depth cutoff was increased from 1 to 10, the average numbers of hetSNPs dropped quickly from 1,321,004 to 30,902; when the cutoff was further increased to 20 and 30, the average hetSNP numbers decreased to 14,925 and 9316 (Additional file 1: Figure S3). The overall declining trend of hetSNP numbers was similar to the pattern in the mouse data (Additional file 1: Figure S1). It should be noted that both the mouse and human studies used the SMART-seq protocol and the Fluidigm C1 system to generate the scRNA-seq data [12, 28]. In the end, we decided to use a read depth of 20, which yielded <0.5% false positive rate based on our analysis of the mouse scRNA-seq datasets (Table 1). Due to different genetic backgrounds and differences in the number of cells analyzed, we obtained variable numbers of hetSNPs for the six individuals (Additional file 1: Figure S4a; Table 1). On average, we obtained 14,925 hetSNPs across the six individuals. For adult50 brains, which had the largest number of cells (77 cells), we got the largest number of hetSNPs (41,465). In contrast, for adult47, which had the smallest number of cells (24 cells), we only identified 3569 hetSNPs. This is expected, as larger numbers of cells sequenced would result in more sites with greater read coverage (Additional file 1: Figure S4a). This also indicates that although genetic backgrounds were different among the six individuals, cell number usage is likely a more important factor for the number of hetSNPs identified from scRNA-seq data. We next analyzed the genomic distribution of these hetSNPs based on the Ensembl gene annotation (release 74). Across individuals, ~80% of the called hetSNPs were located in the genic regions (Additional file 1: Figure S4b), with ~25% in exons. The exonic hetSNPs covered 2193 genes on average, with the maximal of 4413 genes in adult50 and the minimal of 851 genes in adult47 (Table 1).

**Table 1:** hetSNPs and monoallelic genes in human brain cells

Individual	Cell number	Called hetSNPs	Exonic hetSNPs	Genes with exonic hetSNPs	Cell-type MA genes				
					Astrocyte	Microglia	Neurons	Oligodendrocyte	OPC
adult21	57	12,032	3736	2294	0	0	225	76	0
adult37	63	18,101	4072	2491	31	0	358	0	0
adult47	24	3569	1340	851	22	2	7	0	0
adult50	77	41,465	7627	4413	548	0	438	0	0
adult54	58	9996	2612	1667	0	27	0	107	98
adult63B	44	4386	2192	1444	0	0	71	24	0

### Biased expression of functionally disrupted alleles at hetSNP sites

**Fig. 2:**
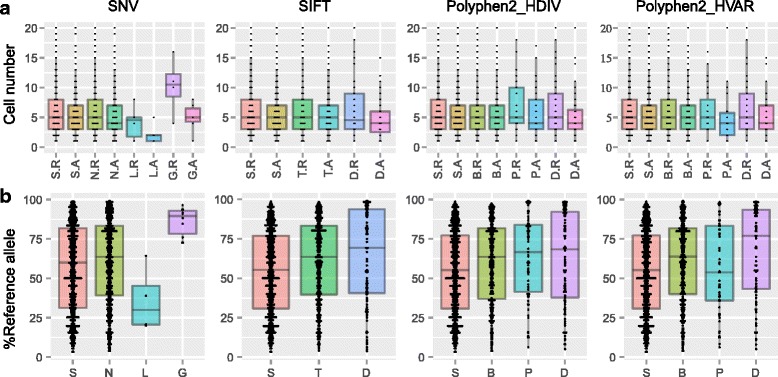
Effects of functionally disrupted alleles on expression in human brain cells. **a** Boxplots showing the numbers of adult21 brain cells expressing reference (R) or alternative (A) alleles (allelic read depth ≥ 2). Each point represents a heterozygous SNP whose classification was derived from the exome summary results by wANNOVAR annotation (see Methods). **b** Boxplots showing the percentages of reference reads (vs total reads) at hetSNPs sites in adult21 brain cells (read depth for each of the alleles was ≥2 and the sum of read depths was ≥10). The SNV classification of hetSNPs: S, synonymous (*n* = 559); N, non-synonymous (*n* = 435); L, stoploss (*n* = 6); G, stopgain (n = 6). SIFT classification: S, synonymous (*n* = 578); T, tolerated (*n* = 340); D, deleterious (*n* = 77). Polyphen2_HDIV classification: S, synonymous (*n* = 587); B, benign (*n* = 387); P, possibly damaging (*n* = 49), D: probably damaging (*n* = 72). Polyphen2_HVAR classification: S, synonymous (n = 587); B, benign (*n* = 315); P, possibly damaging (*n* = 43), D: probably damaging (*n* = 50). As the numbers of functionally disrupted SNPs were small, we also plotted individual points with summary statistics illustrated by boxplots. Note that a SNP expressed in multiple cells would appear as multiple points in panel b. Results for other individuals were in Additional file 1: Figures S5 and S6

To study the potential impacts of allelic gene expression, we first addressed if a functionally disrupted allele would be expressed differently from its counterparts. We predicted the functional impacts of the alternative alleles at hetSNP sites using wANNOVAR [37] and examined if the deleterious alleles were expressed in fewer cells (Fig. 2a; Additional file 1: Figure S5) or at a lower level (Fig. 2b; Additional file 1: Figure S6). Indeed, using the adult21 brain as an example, we found that the alternative deleterious alleles were expressed in significantly fewer cells than the reference alleles, as the medians of expressing cell numbers for the reference and alternative alleles were 13.5 and 5 (*p* = 0.018, one-sided Wilcoxon rank-sum test) for gain-of-stop (G, termed “stopgain” in wANNOVAR) mutations (*n* = 6) (Fig. 2a, SNV annotation). This difference is possibly explained by the fact that the transcripts with a gain of stop mutation would be degraded by the nonsense-mediated mRNA decay (NMD) pathway [38]. The six genes with a stopgain mutation were *IL17RB*, *SBDS*, *DGCR6L*, *SEPT4*, *TMEM14B*, *DFNA5* (Fig. 2a, SNV annotation). Using SIFT annotation, the alternative alleles with predicted functional disruptions were also expressed in significantly fewer cells than the reference alleles: the medians of expressing cell numbers for the reference and alternative alleles were 6 and 4 (*p* = 0.001, one-sided Wilcoxon rank-sum test) for deleterious (D) mutations (*n* = 77) (Fig. 2a, SIFT annotation). In contrast, no large difference was detected at hetSNPs with predicted synonymous changes (S), although a slight bias to reference alleles was observed. Similar results were obtained using the Polyphen2_HDIV and Polyphen2_HVAR annotations (Fig. 2a), and scRNA-seq from other brains (Additional file 1: Figure S5). Taken together, these results indicate that functionally disrupted (alternative) alleles were less abundant in cells than the non-disrupted (either reference or alternative allele) ones, but the mechanisms other than NMD need to be studied in the future.

We then examined if the deleterious alleles were expressed at a lower level. We analyzed hetSNPs with both alleles expressed in the same cell, defined as a read depth for each allele ≥2 and the sum of read depths ≥10 (Fig. 2b; Additional file 1: Figure S6). In general, we observed a slightly biased expression of reference alleles, probably due to technical artifacts, e.g., mapping bias, which remains a big challenge in alignment based analysis [39]. For hetSNPs sites with gain-of-stop (G, *n* = 10) mutations in adult21 brain, the percentages of reads from the reference alleles were significantly higher than 50% and greater than the percentages at sites with synonymous (S, *n* = 677) changes: the medians of reference allele percentages were 89.57% and 60% (*p* = 0.0002, one-sided Wilcoxon rank-sum test; Fig. 2b, SNV), respectively. Similar results were obtained using the predictions from SIFT, Polyphen2_HDIV, and Polyphen2_HVAR annotations, with the reference alleles expressed 15~20% higher at the hetSNPs with deleterious (D in SIFT and P/D in Polyphen2) changes than the hetSNPs with synonymous (S) changes (*p* < 0.00004, one-sided Wilcoxon rank-sum test). Analysis of data from other brains revealed a similar pattern except in the adult47 brain, in which the “deleterious” and “probably damaging” alleles showed similar or lower expression of the reference alleles when compared to synonymous SNVs (Additional file 1: Figure S6). Although the alterative alleles were generally expressed at a lower level than the reference alleles, we found that compared to the alternative alleles leading to synonymous changes, the expression differences between functionally disrupted alleles and the reference alleles were significantly bigger across cells from different brains.

### Allele-biased gene expression in human brain cells

**Fig. 3:**
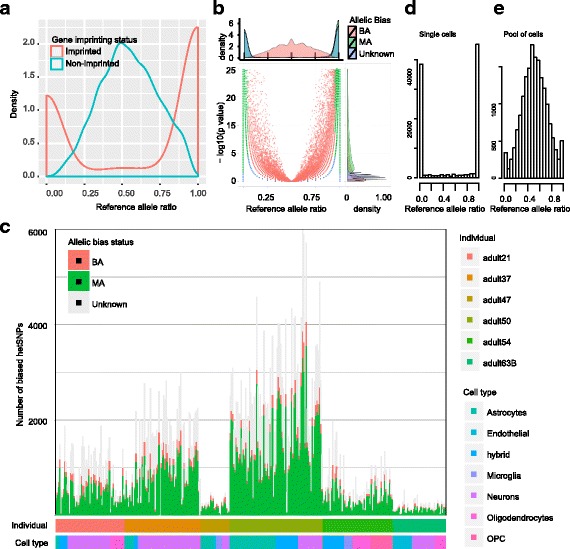
Allele-biased expression of hetSNPs in human brain cells. **a** Distribution of reference allele ratios for imprinted and non-imprinted genes. **b** Distributions of hetSNP frequencies (top) and binomial test *p*-values (bottom) for all hetSNPs broken down by the ratios of reference alleles (x-axis). Data are for all cells and for all hetSNPs in the six adult brains. **c** The distribution of biased hetSNPs in all brain cells grouped by individuals and cell types. **d** Distribution of hetSNPs broken down by reference allele ratios in single neurons of adult37. **e** Distribution of hetSNPs called from pooled scRNA-seq reads from neurons of adult37

We next analyzed the exonic hetSNPs for allele-biased expression. For each of the hetSNPs, we evaluated its allele-biased expression in a single cell by performing a binomial test of the read counts for the two alleles and considering the allelic ratio (see Methods and Additional file 1: Figure S2). To evaluate our method, we first checked the allelic expression of imprinted genes (obtained from Geneimprint: http://www.geneimprint.com/). We found that in pooled RNA-seq reads the ratios of reference expression for imprinted genes were significantly deviated from 0.5, using either mouse or human data (Fig. 3a; Additional file 1: Figure S8). At the single cell level, we analyzed a total of 927 hetSNP sites (the occurrences of the same site in multiple cells were considered independently) in the human imprinted genes and classified 416 as MA, 48 as BA, and 463 Unknown (Fig. 3a; Additional file 1: Figure S7a). The 48 BA occurrences were from 23 unique hetSNP sites in 8 genes (*NTM*, *MEG3*, *MAGI2*, *GNAS*, *MEST*, *DGCR6L*, *PPP1R9A* and *NLRP2*) and they accounted for only a small percentage of total SNPs analyzed in each of these genes (0.8% ~ 13.1%, except for *NLRP2*) (Additional file 1: Figure S7b). Note that BA from imprinted genes could be due to either isoform-dependent allelic expression, e.g., *GNAS* [40], or allelic expression leakage [15]. This result indicates that our criteria for defining allelic expression are reasonably accurate.

To get an overview of the allelic bias status, we first summarized the allelic bias at 284,220 sites in all cells among the six individuals. As expected, the reference allelic ratios of 25,856 biallelic (BA) sites centered around 0.5 (Fig. 3a). For 136,422 monoallelic (MA) sites, the distribution of the reference allelic ratios was bimodal, with ratios near 0 or 1 (Fig. 3a). We also noted that the number of MA sites was approximately 5× larger than that of the BA sites, while 42.90% (“Unknown”) of the tested sites were not well covered for statistical inference at the single cell level (Fig. 3a). We then examined the allelic biased status in each cell (Fig. 3b). Although the numbers of detected hetSNPs varied greatly among cells, the allelic expression patterns were quite similar among all the cells (Fig. 3b). The average percentage of hetSNPs that showed monoallelic and bi-allelic expression is 56.69% and 4.69%, respectively (Fig. 3b), indicating that at a single cell level the majority of genes are expressed from a single allele at a particular time point, consistent with recent findings [12, 26, 31, 32]. To address how frequently two single cells shared MA alleles, we randomly chose two neurons from adult37 and calculated the percentages of shared MA alleles between two neurons. Repeating this process 1000 times we found that the percentage of shared MA alleles was 9.50% on average (1.82% ~16.88%), indicating a high degree of cellular heterogeneity and/or technical noise.

To see how allelic expression in single cells would be reflected at the cell population level, we pooled scRNA-seq data from the same cell types of the same individuals and then called allelic expression from the pooled scRNA-seq reads (to mimic bulk RNA-seq analysis). Taking neurons in adult37 as an example (Fig. 3c, d), which had the largest number of cells for a specific cell type in any of the six individuals (Additional file 2: Table S1), we found that at the single cell level for 83.87% of the 99,723 total hetSNPs, the reference allele ratios were near 0 or 1 (i.e., strongly MA) in the 50 cells. However, when scRNA-seq reads from the 50 neurons were pooled and analyzed as bulk RNA-seq data, we found that 6484 (36.93%) of the 17,559 non-redundant hetSNPs showed reference allele ratios between 0.4 and 0.6 (Fig. 3c, d). Taken together, these results indicate that the paternal and maternal alleles were randomly expressed in a highly biased manner in individual human neurons. The same analysis for other cell types showed that as scRNA-seq data from more cells were pooled, more hetSNP sites exhibited biallelic expression (Additional file 1: Figure S9 and S10). These results indicate that both alleles of a gene can be expressed but only one is predominately expressed in a single cell and the choice is mostly random. This finding is consistent with previous observations [12, 32] and suggests that allelic expression in human brains is not much different from other tissues.

### Biased gene expression in individual human brain cells

**Fig. 4:**
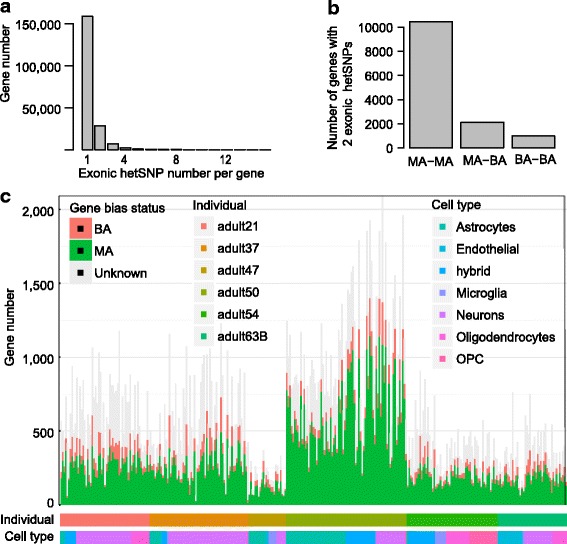
Biased gene expression in human brain cells. **a** Distribution of genes with different numbers of exonic hetSNPs. This is a summary of all cells in the six individuals. **b** Comparison of allelic expression for genes with exactly two exonic hetSNPs. **c** Distribution of the numbers of genes with three patterns of biased gene expression across individual cells

To study the potential functional impact of allele-biased expression, we mapped the hetSNPs to genes in each cell. There were 257,167 non-unique (i.e., redundant) hetSNP sites in total when all the cells were considered together, and they were mapped to 198,690 genes in total (a gene might be counted more than once if it was expressed in many cells) (Additional file 3: Table S2). 158,899 out of the 198,690 genes (79.97%) had only one hetSNP detected by scRNA-seq from a particular cell, while 28,688 (14.44%) harbored two hetSNPs and 11,103 (5.59%) contained >2 hetSNPs (Fig. 4a). One way to evaluate the accuracy of our allelic expression results is to check the self-consistency between two hetSNPs within the same gene. Among the 13,570 genes harboring two hetSNPs, 11,439 (84.30%) genes showed consistent biased expression, either both were monoallelically (MA-MA) or both were biallelically (BA-BA) expressed (Fig. 4b), supporting that our calls for allelic expression were accurate. We also compared the expression level of the three groups of genes and found that genes of BA expression were overall expressed at a higher level (BA-BA vs. MA-BA: Wilcoxon rank sum test, *p* value = 0.007; MA-BA vs. MA-MA: Wilcoxon rank sum test, p value <2.2e-16) (Additional file 1: Figure S11a,b).

After the hetSNPs were mapped to genes, we determined the allelic expression status of all the expressed genes for each cell (Additional file 1: Figure S2). We considered a gene to be bi-allelically expressed if any of its hetSNPs was BA, or otherwise monoallelically expressed if it contained a MA hetSNP. In the end, we found that the average percentages of genes exhibiting MA and BA expression were 50.24% and 10.08%, respectively (Fig. 4c).

### Biased gene expression in individual human brain cell types

A human brain is made up of a heterogeneous mix of cell types, each performing their unique functions. We thus asked how allelic gene expression differed among cell types. We began by identifying genes that exhibited MA (or BA) across multiple cells of the same cell type. As a previous study raised the concern that low expressed genes were more likely to be called monoallelic expression due to technical limitations in the scRNA-seq assay [12], we restricted our analysis to genes that were expressed at the top 30th percentile level in each cell, after excluding non-expressed genes. The cutoff values for the top 30th percentile of genes in the cells were 24.8 FPKM on average, but they varied among cells (Additional file 1: Figure S12). To test if we could get consistent MA genes for a certain cell type from the available cells, we randomly split the 50 neurons in adult37 into two groups (25 cells each) 1000 times and calculated the percentages of overlapping MA genes. The mean percentage was 27.78% (min 20.22%, max 35.71%). As the number of cells became a factor in assessing allelic expression at the level of cell type, we evaluated its influence using again the 50 neurons from adult37 brain. We randomly sampled a subset of the 50 cells (from 1 to 49) and then determined the number of allelically expressed genes by our method. We repeated the process 1000 times (or used all possible combinations when fewer than 1000) and found that the number of MA genes continually increased as the number of neurons used for analysis increased, indicating that the 50 neurons were still insufficient to identify all MA genes in this cell type (Additional file 1: Figure S13). A close examination of this issue found that for most genes their MA statuses were shared in only a few cells (Additional file 1: Figure S14). For example, in adult50 neurons, 2160 out of the 3488 MA genes (61.93%) were called MA in less than four cells. Likewise, in adult54 microglia, 526 out of 565 MA genes (93.10%) were evaluated as MA in less than four cells.

Nevertheless, we reasoned that a gene needs to exhibit MA “consistently” if its MA expression would confer any functional affect to a brain cell type, so we analyzed the genes that were called MA expression in at least four cells of the same type, with no cell exhibiting a BA pattern (Additional file 1: Figures S2 and S14). By this definition, we obtained 145 MA expressed genes on average in the five brain cell types of six individuals, with the most (*n* = 548) in astrocytes from the adult50 brain and the least (*n* = 2) in microglia from the adult47 brain (Table 1; Additional file 4: Table S3). When we took the total hetSNPs called for an individual into consideration, on average 5.37% of the heterozygous genes in the six individuals showed strong biased allelic gene expression in one of the six brain cell types (Table 1). To evaluate our list of MA genes, we merged our lists of MA genes from all cell types and all individuals and checked them (1515 unique genes in total) against the database of human and mouse autosomal monoallelic genes – dbMAE, which contained two broad classes of data: direct measurement of allelic expression imbalance (termed ‘experimental’) and indirect chromatin-based inference (‘inferred’) [41]. We found that 688 of our 1515 MA genes were present with experimental evidence, among which 65 genes (9.45%) were biased in at least one of the eight human tissues recorded in the dbMAE. We also found that among 1256 genes with inferred evidence for MA, 468 genes (37.26%) showed biased expression in at least one human tissue (Additional file 4: Table S3). We also compared our list against the mouse data in dbMAE. We found 1214 MA genes with experimental evidence, among which 712 genes (58.65%) were biased in at least one mouse tissue, and 1213 genes with inferred evidence, among which 562 genes (46.33%) were biased in at least one mouse tissue. This comparison shows that there is a broad agreement between our calls and the allelic expression reported in the dbMAE.

### Functional analysis of biased genes in human brain cell types

**Fig. 5:**
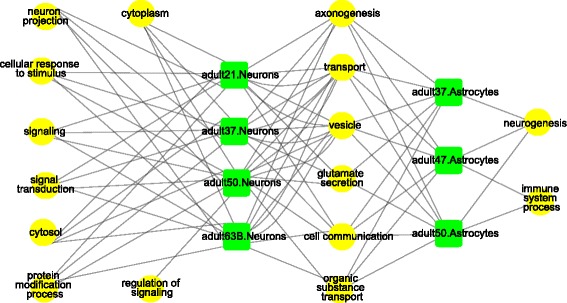
Functional analysis of MA expressed genes based on GOseq. The yellow round nodes stand for the representative terms of enriched functional groups. The green square nodes stand for cell groups, corresponding to cells from the same cell type in an individual. An edge was used to link an enriched GO term to a cell group, if the percentage of MA genes with the enriched terms in the cell group is ≥ the percentage of MA genes with the same term for all groups

After we obtained the small lists of MA genes for different cell types (Additional file 4: Table S3), we first studied their functions separately for each cell type using the software GOseq [42] (Fig. 5). Genes with expression levels similar to MA genes, i.e. the top 30% in expression level in each cell of the same cell type, were combined and used as background genes. We did not obtain significantly enriched terms (adjusted *p* value <0.05) in oligodendrocytes, OPC, and microglia, possibly due to the small numbers of MA genes for those cell types (Additional file 4: Table S3). To determine if the MA genes from astrocytes and neurons in different individuals showed similar functions, we used a network to illustrate the relationship between groups of cells of the same cell type in different individuals and enriched GO terms (see Methods) (Fig. 5). For neurons, GO terms shared across the MA genes among individuals were neuron projection, signal transduction, and several others (Fig. 5). For astrocytes, the shared GO terms include neurogenesis and immune system process, consistent with its role in regulation of neurogenesis [43] and immune response in the CNS [44]. There were also terms shared by astrocytes and neurons such as cell communication (Fig. 5).

In addition to function enrichment analysis, we also checked against the risk genes for autism [45] and schizophrenia [46]. Although we found that 50 and 67 of the MA genes have been implicated in autism and schizophrenia, the lists of MA genes in the brain cell types as a whole showed no significant enrichment for either autism or schizophrenia (Fisher’s exact test, *p* = 0.33 for autism, *p* = 0.83 for schizophrenia) (Additional file 2: Table S4). Nevertheless, there are several notably interesting genes, including *GRIA3*, *GRIK2*, *NRXN1*, and *NRXN3* (Additional file 2: Table S4). In addition, 20 of our brain MA genes, including autism risk genes (e.g., *ANK2*, *NF1*) and schizophrenia risk genes (e.g., *APC*, *EGR1*, *FGFR3*, *PMP22*, *TCF4*, *TFRC*, and *YWHAE*), were also included in the database of human haploinsufficient genes [47] (Fisher’s exact test, *p* = 0.54; Additional file 2: Table S4), suggesting that some of our MA genes may be quite susceptible to damaging mutations that could lead to a loss of gene expression in subsets of brain cells.

### Cell type-specific monoallelic gene expression in human brains

**Fig. 6:**
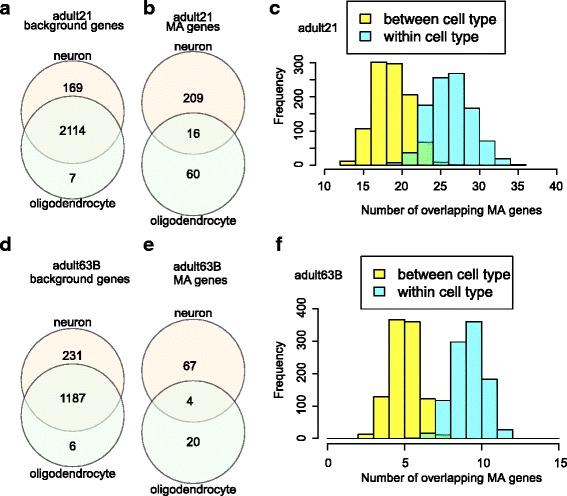
Cell type-specific expression of MA genes. Venn diagrams (**a** and **b**) show the overlaps of background and MA genes between two cell types (35 neurons and 12 oligodendrocytes) from adult21 brains. In histogram **c**, two non-overlapping groups of 12 neurons were randomly sampled 1000 times from the 35 adult21 neurons and the numbers of overlapping MA genes between the two groups was shown in light blue histogram (within-cell type comparison); the numbers of overlapping MA genes between 12 sampled neurons and the 12 oligodendrocytes in adult 21 brains was shown in yellow histogram (between-cell type comparison). The mean values of the two groups are significantly different (t-test, *p* < 2.2e-16). Panels **d**, **e** (18 neurons and 9 oligodendrocytes) and **f** (t-test, p < 2.2e-16) are for adult63B brains

To address if the MA genes were cell type-related, we compared the MA genes between cell types. To obtain meaningful results, we restricted our analysis to individuals with relatively large numbers of cells in two or more cell types. We first compared MA genes in neurons and oligodendrocytes from adult21, with 35 and 12 cells analyzed by scRNA-seq, respectively. There were 2283 and 2121 genes with expressed hetSNPs in neurons and oligodendrocytes respectively, among which 2114 genes (92.31%) were shared between the two cell types (Fig. 6a). However, there were only 16 MA genes (5.61%) shared by the two cell types (Fig. 6b), indicating that most of the MA genes were cell type-specific. We then repeated this analysis using brain data from another individual – adult63B, and obtained a similar result with 1187 background genes (83.36%) shared but only 4 MA genes (4.40%) shared between neurons and oligodendrocytes (Fig. 6d,e). Comparisons of MA genes in another two cell types, astrocytes and neurons, from three individuals (adult37, adult47 and adult50) uncovered a similar trend (Additional file 1: Figure S15). To address if the small overlap was due to small and unequal numbers of cells being analyzed and thus likely technical artifact, we performed permutation tests. For adult21 data (Fig. 6c), we randomly sampled two sets of 12 cells (no cells common between the two sets) from the 35 adult21 neurons 1000 times and obtained the frequency of shared MA genes between the two sets of neuronal cells. We also randomly selected 12 cells from the 35 adult21 neurons and iterated the analysis 1000 times; overlap was computed for the MA genes between the random neuron set and the 12 oligodendrocytes. The result shows that the numbers of overlapping MA genes between cell types (neurons vs oligodendrocytes) was significantly smaller than that obtained from intersecting two sets of the same cell type (neurons). Repeating this analysis using scRNA-seq data from adult63B yielded similar results (Fig. 6f). The same permutation tests were also done with astrocytes and neurons in three individuals, yielding the same conclusions. (Additional file 1: Figure S15). Taken together, these results support the idea that MA expressed genes in human brains are generally cell type specific, an intriguing observation to be further explored with more cells.

We found some shared MA genes in the same cell types across individuals. For example, 86 of the 1006 MA genes in neurons showed MA expression in at least 2 individuals. Among them, *PCDH9* exhibited MA expression in 4 individuals; *PCDH9* is a member of the cadherin superfamily of calcium-dependent cell adhesion molecules and was previously reported to show monoallelic expression [4, 5]. In Oligodendrocytes, 11 of the 196 MA genes showed MA expression in at least 2 individuals. There were no shared MA genes across individuals for microglia (29 MA genes in total) or OPC (98 MA genes in total). The reason for the small overlap of MA genes is explained by the scarcity of shared expressed hetSNPs among individuals.

### Co-expression of monoallelic genes in neurons

**Fig. 7:**
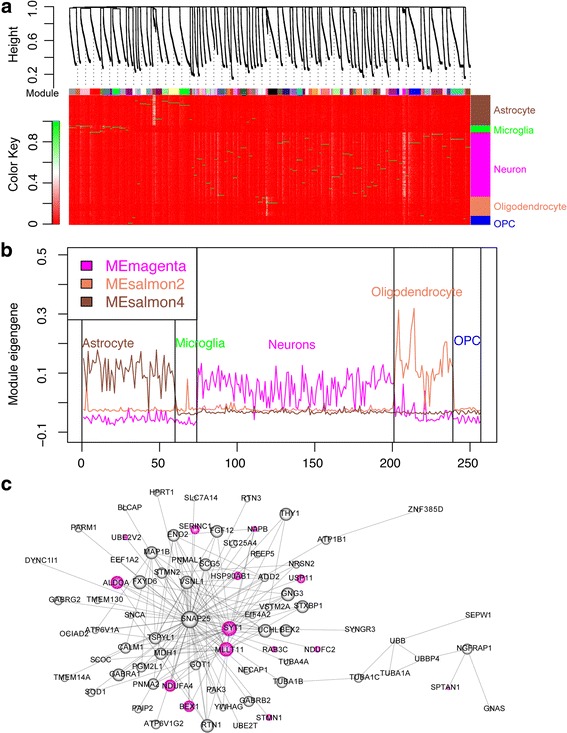
Co-expression of MA genes. **a** Dendrogram shows the WGCNA modules and the heatmap shows the gene expression of module genes in different samples. **b** Eigengene expression of three selected modules in all samples. The three modules correspond to three cell types: astrocyte, neuron and oligodendrocyte. No modules for microglia or OPC were identified. **c** Network shows co-expression of genes in the neuron module. Node sizes correspond to intramodular connectivity. MA genes were colored magenta, the same as the module color

Next, we studied if MA genes were co-expressed by WGCNA analysis [13, 48]. Using the scRNA-seq data from all 323 cells, we performed a WGCNA analysis [49] and identified 181 co-expression gene modules. We found that the magenta module showed the highest expression only in neurons, while the salmon2 module and the salmon4 modules exhibited the highest expression in oligodendrocytes and astrocytes, respectively (Fig. 7a; Additional file 2: Table S5). We did not observe modules that were especially highly expressed in microglia or OPC. The expression profiles of the eigengenes for the three modules also supported the idea that these modules were highly expressed in only one particular type of cell (Fig. 7b). GO analysis showed that the 34 genes in the salmon2 module were enriched for axon ensheathment and central nervous system myelination. The 52 genes in the salmon4 module were enriched for astrocyte differentiation functions. The 147 genes in the magenta module were enriched for synaptic transmission, regulation of membrane potential, and Alzheimer’s disease. We further examined the module genes and found that they contained cell type-related marker genes (Additional file 2: Table S5). For example, neuron marker genes, such as *TMEM130* [50], *MAP2*, *MAP1B*, *SNAP25*, *PGM2L1* and *SCG2* [51], were in the magenta module; oligodendrocyte marker genes, such as the mature oligodendrocyte marker *MBP* [51, 52], *CLDN11* [53], *OPALIN* [54], *ERMN* [55], *PLP1*, *HSPA2*, *MOG* and *PPP1R14A* [51] were in the salmon2 module; and astrocyte marker genes, such as *AQP4* [56], *ATP1A2*, *ALDOC*, *SLC1A2*, *GLUL* and *AHCYL1* [51] were in the salmon4 module. This result indicates that the three modules are possibly cell type-related modules containing genes that could potentially serve as marker genes for the respective cell types.

We then examined if MA genes were enriched in these three modules. Interestingly, we found that MA genes in neurons of adult50 were enriched in the magenta module (Fisher exact test, *p* value = 2.9E-3) (Fig. 7c), which was highly expressed in neurons. Analyzing data from neurons of individuals with different ages, we found 13% of the MA genes in adult50 neurons also showed MA expression in at least one of other samples, indicating that some MA genes may function at different developmental stages. Surprisingly, five hub genes, *SYT1, STMN1, NGFRAP1, NAPB* and *BEX1*, were identified as monoallelically expressed. SYT1, which showed MA expression in neurons from three individuals, is a synaptic vesicle integral membrane protein thought to serve as a Ca^2+^ sensor in vesicular trafficking and exocytosis. Calcium binding to SYT1 protein participates in triggering neurotransmitter release at the synapse [57]. *STMN1*, showing MA expression in two individuals, is a neuronal growth associated protein that is involved in microtubule dynamics and plays an important role in synaptic plasticity and neurite outgrowth [58]. *NGFRAP1* (also known as BEX3), identified as a MA gene in neurons of two individuals, is involved in regulating NGF-dependent neuronal survival and differentiation [59]. The *NAPB* gene encodes a cofactor involved in soluble N-ethylmaleimide-sensitive fusion attachment protein receptor (SNARE)-complex-dependent synaptic vesicle fusion and recycling (synaptic vesicle docking) [60] *Bex1* is involved in the regeneration of axons after injury [61] and serves as an interactor of the p75 neurotrophin receptor, linking neurotrophin signaling to the cell cycle [62]. It will be interesting to determine whether the monoallelic expression pattern of these genes in neurons plays a role in diversifying synaptic activity.

## Discussion

Monoallelic gene expression, such as imprinting, X-chromosome inactivation, and selective expression of immune response genes and olfactory receptor genes, has been known for decades. The two alleles of a gene can also be expressed differently if genetic mutation(s) disrupts the regulatory regions in one of them specifically, rendering one allele to be expressed at a lower level or not at all. The application of massively parallel transcriptomic technologies, either microarray or RNA-seq, has revealed that for most human and mouse genes the two alleles are frequently expressed in a biased manner, largely due to genetic variation [63, 64]. While most of the previous studies were performed in cell lines or stem cells, in this study, we re-analyzed scRNA-seq data of adult brain cells and found that at the single cell level most of the genes show allele-biased expression, indicating that monoallelic expression seems to be the norm rather than exception. Our finding is consistent with recent in vitro studies [20, 22] and indicates that neurons and other cell types in the brain all display widespread monoallelic gene expression at the single cell level. In addition, based on bulk RNA-seq analysis, the GTEx project has also studied the allelic expression across human tissues and found that the proportion of shared MA genes between tissues varies from 0.85% to 39% (mean 11%), suggesting substantial tissue specificity [64]. Although we did not find a big difference among brain cell types in terms of the extent of monoallelic gene expression, we showed that some MA genes were expressed monoallelically in specific brain cell types and MA genes in oligodendrocytes and neurons were involved in cellular functions specific for them. These findings suggest that some deleterious heterozygous mutations may affect particular cell types more than others, adversely affecting brain development by disrupting different cellular components of the brain.

Identification of MA genes from scRNA-seq data is a challenging task and needs more studies at the levels of both data collection and algorithm development. Many tools have been successfully developed for allelic gene expression from bulk RNA-seq data. For example, a meta-analysis based allele-specific expression detection for ASE expression (MBASED) works quite well by aggregating information across multiple SNPs of the same gene [65]. In scRNA-seq, for most cases, however, only one SNP of a gene has sufficient coverage in a sample and the same SNP is rarely covered across multiple samples. Several studies have mentioned that the technical allelic dropout in scRNA-seq could inflate the observation of MA expression [12, 31]. Dynamic transcriptional burst can also result in a “failure” in capturing both alleles in scRNA-seq [12]. Considering these possibilities, we have taken a strategy that would call BA genes favorably. Moreover, we only studied genes whose expression was ranked at the top 30% in the analysis of cell type allelic expression, to reduce the possibility for false identification of MA genes, because it was found that genes with higher expression levels were less prone to show a false pattern of MA expression [12, 31]. The reasons are both technical and biological. Firstly, it is easier to capture both alleles in scRNA-seq for higher expressed genes; secondly, more highly expressed genes may have a greater probability of being activated from both alleles, and thus both alleles are present in the cell at any given time [16]. Of the genes with hetSNPs, we found 5.37% on average in the six individuals showed MA expression at the cell-type level (Table 1). This is smaller than what has been reported in previous studies. Using microarray analysis, 10–15% of autosomal genes were found monoallelically expressed in clonal populations of human and mouse lymphoblastoid cells [66]. A scRNA-seq analysis on mouse embryonic cells showed that 12–24% of autosomal genes were monoallelically expressed across the pre-implantation developmental stages [12]. Much of the difference could be due to the different definition of allelic expression (i.e., monoallelic vs allele-biased), but the small number of cells used in our scRNA-seq datasets may be a key reason behind the difference, as discussed above (Additional file 1: Figure S13). Allelic gene expression from scRNA-seq data is an active research area, with new algorithms being constantly released and improved. Some recent developments include the usage of combining Fisher’s exact test with expression threshold to dissect clonal and dynamic monoallelic expression [15] and SCALE, or Single-Cell ALlelic Expression for examining allele-specific transcriptional bursting kinetics [67]. As shown in Figs. 3 and 4, a large portion of hetSNPs and genes were marked as allelic expression “unknown” due to insufficient read coverages for statistical analysis. New experimental procedures should be developed in the future to capture the lowly expressed genes that were either excluded or marked as “unknown” in the current analysis in order to find out if they are indeed subject to the same level of allelic expression as the highly expressed genes.

Previous studies of in vitro neural stem cells or neurons showed that MA genes are enriched in neuroactive ligand-receptor interactions and extracellular interactions [20], and neurodevelopmental disorders such as autism and schizophrenia [14, 19, 21]. Our analysis of cells derived from human brains showed that MA genes are enriched for functions closely related to individual cell types (Fig. 5). For example, MA genes in astrocytes are enriched in neurogenesis [43] and immune system process [44]. We also observed that 50 and 67 genes exhibiting MA expression in multiple cells of the same cell types were implicated in autism and schizophrenia, but the overlap is statistically insignificant.

Overall, we did not observe a significant overlap of MA genes between different brain cell types, indicating that MA expression is likely cell type-specific in in vivo brain cells (Fig. 6). In a previous study on ASD patients and controls, the authors found that the monoallelic expression of several genes (found in two patients) was confined to specific brain regions or cell types [68]. Our finding is also in line with a recent study of allelic expression in developing brains [26]. The tissue- or cell type-specific MA expression patterns suggest that there may be tissue or cell type-specific transcription regulators that can activate one allele while repressing the other. Although the mechanism remains unclear, genes coding for olfactory receptors [3] and protocadherins [4, 5] are known to be expressed in a monoallelic manner in individual neurons. Once monoallelic expression is established, descendant cells can inherit the pattern by epigenetic mechanisms, such as differential DNA methylation or histone methylation in the two parental alleles. In fact, a comprehensive study of DNA methylation in 18 human tissues from 4 post-mortem individuals showed that allele-specific methylation is well correlated with allele-specific expression [69]. An independent study also showed that monoallelic DNA methylation could be associated with some genes, though no common feature could be identified to account for this remarkable epigenetic stability of MA expression [20]. Histone methylation of H3K4 (H3K4me2 and H3K4me3) and H3K9 (H3K9me3) was associated with active and inactive alleles, respectively [22], but H3K27me3, a mark for repressed genes, was surprisingly not associated with inactive alleles [22]. Up to now, there is still no single epigenetic mark that can explain the maintenance of MA expression except imprinting and X-linked inactivation [16]. One possible reason is that the cellular memory at different MA loci may rely on a combination of epigenetic marks or a variety of mechanisms including some still to be discovered. Recently, investigators have begun to understand gene regulation from the perspective of the 3D genome, which refers to gene expression changes caused by inter- and intra-chromosomal interactions. Both Hi-C and ChIA-PET data demonstrate that the 3D organization of the genome shows cell type-specific patterns [70–73]. Similar to the intensively studied CpG methylation, mCH, the newly discovered non-CG methylation [69, 74] also shows a cell type-specific pattern [69]. Both 3-D genome organization and mCH could be novel perspectives to study the regulation of MA expression. In summary, monoallelic expression can occur in a cell type-specific manner but the underlying epigenetic mechanisms for its stable inheritance remain unclear.

Our co-expression analysis identified gene modules that are actively expressed in individual cell types (Fig. 7), indicating that brain cell types can be distinguished by their gene expression signature, and also confirming the classification of brain cell types by the original authors [28]. In addition, the result expanded the cell type-enriched gene list beyond the few known gene markers used in the original study. When comparing our cell type-specific MA genes with the cell-type WGCNA modules, we found that neuron MA genes were enriched in the magenta module, which is involved in various neural functions, such as synaptic transmission and neuron projection. It is conceivable that genes in this module may allow more diverse response to neurotransmitters among neurons. On the other hand, monoallelic expression could also increase disease susceptibility, conceivably, if one copy of the MA gene possesses deleterious mutations such that its expression or lack of expression leads to abnormal function of a specific brain region derived from the clonal expansion of a precursor in which MA expression first occurred. This is consistent with the finding that ~50% of monozygotic twins are discordant for schizophrenia [14].

There are several limitations in our current study. First of all, the genotypes for all individuals are unknown. Our method of deriving hetSNPs from RNA-seq data will miss hetSNPs that express only one of the two parental alleles across all cells in an individual. Secondly, without phasing the hetSNPs, for the MA genes with more than one SNP, we could not accurately tell whether biased SNPs at different sites originated from the same parental allele. Thirdly, the number of cells for some cell types was small, making it hard to identify monoallelic expression at the cell-type level. Fourthly, we could not tell fixed monoallelic expression from dynamic monoallelic expression due to lack of cell lineage information for isolated brain cells. Finally, due to technical limitation in capturing reads from very small amounts of RNA in a cell, there is much noise in scRNA-seq data, especially for low expressed genes. In addition, despite masking the hetSNPs, our method could not totally overcome the inherent reference bias problem in alignment-based data analysis, an area under active investigation. One potential reason is the presence of private SNPs in individuals that are in linkage disequilibrium with the masked hetSNPs. We hope these limitations will be reduced when we apply our analysis to scRNA-seq datasets containing thousands of cells, as they become available.

## Conclusions

In this study, we re-analyzed the human brain scRNA-seq data from the perspective of allelic gene expression, which is different from the original study, and found monoallelic gene expression is prevalent in human brain cells, which may play a role in generating cellular identity and neuronal diversity and thus increasing the complexity and diversity of brain cell functions. We demonstrated that the accumulating scRNA-seq datasets are invaluable resources for further re-exploration. We also pointed out some problems encountered during our analysis, which may help other researchers to better their experimental designs on allelic expression research using scRNA-seq.

## Methods

### Datasets

Two scRNA-seq datasets were downloaded from the GEO database. The dataset of human brain cells (Additional file 2: Table S1, GEO accession: GSE67835) classified adult brain cells into astrocytes, microglia, neurons, oligodendrocytes and oligodendrocyte precursor cells (OPCs) [28]. We used the authors’ original classification. The dataset of mouse embryos (GEO accession: GSE45719), in which 42 F1 embryos of two mouse strains, CAST/Ei and C57BL/6, at 10 stages, including zygote, early 2-cell, middle 2-cell, later 2-cell, 4-cell, 8-cell, 16-cell, early blast, middle blast and later blast stages, were used to determine the expression of maternal and paternal alleles [12]. We used this dataset to calibrate our hetSNP calling method.

### Identifying hetSNPs from scRNA-seq data without genotyping data

In order to study the different expression of two alleles, we first need to identify genes with heterozygous SNPs. There are software programs that can call SNPs from bulk RNA-seq datasets, such as GATK [35], samtools [75], and eSNV-detect [76]. However, they usually do not work as well on RNA-seq data as they do on genomic sequencing data, because the assumption of a 1:1 ratio of the two parental alleles are often violated in RNA-seq data, resulting in increasing errors (see discussions in http://gatkforums.broadinstitute.org/gatk/discussion/3891/calling-variants-in-rnaseq). They are therefore especially not suitable for our study. As such, we adapted a more straightforward SNP calling method for bulk RNA-seq to scRNA-seq [33, 34]. The method uses known SNPs in the dbSNP that are polymorphic in general human population, computes RNA-seq read coverage for the two alleles, and then evaluates heterozygosity (Fig. 1a). To do this, we pooled the scRNA-seq data for cells from the same individual and then analyzed reads covering candidate SNP sites that were reported in the dbSNP database (for human, dbSNP version 142 downloaded from UCSC) or the mouse genome resequencing project [77] (version 5, ftp://ftp-mouse.sanger.ac.uk/REL-1505-SNPs_Indels/mgp.v5.merged.snps_all.dbSNP142.vcf.gz). We first masked these SNP sites with “N” in the human genome (hg19) or mouse genome (mm10) and then aligned pooled scRNA-seq reads to the modified genomes by STAR (ver. 2.4.2a) [78], allowing 4% mismatches at most (−−outFilterMismatchNoverLmax 0.04), or 2 mismatches in the 50 bp reads. Since the multi-mapped rates were low, only uniquely mapped reads were kept for later analysis (−−outFilterMultimapNmax 1) to reduce ambiguity. Duplicate reads were removed using samtools (ver. 0.1.19) with default settings. Next, we used the samtools mpileup command to obtain allelic read depth at the candidate sites that were masked. For mouse embryonic data, we extracted a list of hetSNPs (*n* = 17,491,332, 0.67% of the mouse genome) that are different between the two parental mouse strains (CAST/Ei and C57BL/6). We then called hetSNPs from the scRNA-seq data. We considered a site heterozygous if each of the two alleles was supported by a minimal number of reads (read depth cutoff), which was tested from 1 to 30. The resultant hetSNPs were then checked against the known genotype derived from the mouse genome project to evaluate the accuracy of our SNP calling (Additional file 1: Figure S1). Based on the mouse data, we determined that for human brain cells, a site could be confidently scored as heterozygous if both alleles were supported by ≥20 reads.

### Identifying monoallelic genes from scRNA-seq of human brain cells

After testing our SNP calling method on mouse data, we applied it to human brain cells. If neither of the two alleles was the reference allele, the SNP position was excluded from further analysis. Only a few such positions (min 5, max 92) were observed in the samples. After a list of hetSNPs was called for each individual from the pooled scRNA-seq data, to get an overview of the genomic distribution of the hetSNPs, we first annotated the hetSNPs based on Ensembl gene annotation (Release 74), which contains 63,677 genes including 22,810 protein-coding and 56,337 non-coding genes. A hetSNP would be excluded from further analysis if it is mapped to more than one gene. We predicted the functional impacts of all hetSNPs using wANNOVAR and specifically analyzed the “exon summary results” from wANNOVAR [37], which separated SNPs into synonymous (S), non-synonymous (N), stoploss (L) and stopgain (G) mutations. For non-synonymous mutations, we further used the SIFT scores and PolyPhen scores from wANNOVAR to identify the deleterious (D) or tolerated (T) mutations (Two databases, HVAR and HDIV, were used), and to classify the “probably damaging” (D), “possibly damaging” (P) and “benign” (B) mutations. Then, we compared the numbers of cells and expression level between reference and alternative alleles across SNPs in different categories. In this analysis, an allele covered by at least two reads was considered expressed in a cell. After that, we analyzed allele-biased expression of these SNPs in each cell. The data processing procedure is illustrated in a supplementary figure (Additional file 1: Figure S2). To determine the allelic expression pattern for each hetSNP, a binomial test was applied with *p* values adjusted (FDR) by the BH method, and an allelic ratio was calculated [14, 22, 79]. The hetSNP sites were considered to show a monoallelic (MA) expression pattern, if the FDR was <0.05 and >95% reads were from one allele, similar to what was described previously [15]. Even though 95% was a very strict bias cutoff, the application of binomial test was necessary; otherwise misclassification could occur to a true BA expressed hetSNP with very low allelic read coverage and all reads (e.g., 5) from one allele. To reduce false calls, we only considered a SNP site to show biallelic expression (BA) if it did not satisfy the MA criteria and both alleles had at least two reads to confirm their expression [12]. The allele expression pattern of the remaining hetSNPs (with at least one read) was classified as “Unknown.”

To map the SNP-level biased expression to gene-level biased expression in each cell, we considered the biased status (MA and BA, excluding “Unknown”) of all the hetSNPs in the exons of a gene in a hierarchical manner. A gene was considered to show BA expression in a cell if any of its hetSNPs was assessed as such. However, if only a MA pattern was observed for its hetSNPs, this gene would be regarded as MA in a cell.

After assessing the biased expression states of each gene in each cell, we compared a gene’s biased expression across cells of the same cell type (from the same individual) to evaluate cell-type biased expression. Previous studies showed that lowly expressed genes tended to be misidentified as MA genes. Deng et al. found that the allelic losses are a function of the expression level with low expressed genes showing a high rate of allelic losses [12]. Another report using external RNA spike-ins also demonstrated that low expressed genes frequently display stochastic monoallelic expression which is unlikely to be genuine [31]. To minimize the effect of gene expression level on gene bias decision, especially for low expressed genes, we classified a gene as BA expression in a cell type if it was called BA in any cell of the cell type. For MA expression in a cell type, we set the following criteria: (1) all cells (at least four) support the MA expression in the cell type; and (2) in each cell, the gene expression level should rank at the top 30% by expression level [12].

### Function analysis of MA genes

We performed a gene ontology enrichment analysis of all MA genes using GOseq, which corrects the over-detection of long and highly expressed transcripts in enrichment analysis [42]. Both the Biological Process and Cellular Component ontologies were used for the enrichment analysis. *P* values from GOseq were further adjusted using BH method implemented in the R function p.adjust. Only terms with adjusted *p* value <0.05 are shown. GO terms with too general meanings (e.g. “GO:0044464:cell part”) were not shown in results. To compare enriched GO terms across cell types, we created a GO-term network. We first took genes that were called MA in any cell type and used GOseq to identify enriched terms. An edge was used to link an enriched GO term to a sample (a group of cells from a specific cell type in an individual were treated as a sample), if the percentage of MA genes with the enriched terms in the sample is ≥ the percentage of MA genes with the same term for all samples. The network with samples was reproduced in Cytoscape 3.2.0. MA genes were compared to databases of disease candidates, i.e. SzGene [46] and ASD candidates [45], to find enrichment.

### Comparison of MAs between cell types

To compare MA expression between cell types statistically, we used the Fisher’s exact test and the expressed genes (mean FPKM ≥1 in the cell type) with hetSNPs in each cell type as the background genes. To get an empirical statistical significance, we randomly sampled the same number of cells between and within cell types 1000 times, identified the MA genes in each sampling set, and compared the overlaps between and within groups.

### Enrichment of MA genes in WGCNA module

A signed network was constructed using genes with average FPKM value ≥1 in cells expressing that gene [49]. Missing entries and zero-variance genes were removed. Soft-thresholding power for network construction was estimated and used to derive a pairwise distance matrix for the remaining genes using the topological overlap measure. The intramodular connectivity, i.e. connectivity of nodes to other nodes within the same module, was used to evaluate the “hub-ness” of a gene within each module. The closest 150 edges of a module with MA genes enriched were exported to Cytoscape for visualization.

## Additional files

Additional file 1: Figures S1–S15. **Figure S1.** SNP calling result using mouse embryonic scRNA-seq data. **Figure S2.** A cartoon illustrating the steps and criteria in our allelic expression. **Figure S3.** Numbers of hetSNP called for the six human brains. **Figure S4.** The effect of cell numbers on hetSNP calling and the genomic distribution of hetSNPs. **Figure S5.** Boxplots showing the numbers of brain cells expressing reference (R) or alternative (A) alleles (allelic read depth ≥ 2). **Figure S6.** Boxplots showing the percentages of reference reads (vs total reads) at hetSNP sites in brain cells (read depth for each of the alleles was ≥2 and the sum of read depths was ≥10). **Figure S7.** Allelic expression of hetSNPs within human imprinted genes in brain cells. **Figure S8.** Allelic expression of hetSNPs within mouse imprinted genes in embryonic cells. **Figure S9.** Numbers of hetSNPs sites with different reference allele ratios. **Figure S10.** Numbers of hetSNPs sites with different reference allele ratios, after scRNA-seq reads from cells of the same type in individual brains were pooled. **Figure S11.** Statistical summaries of allelic expression at the gene level. **Figure S12.** FPKM cutoff values for defining the top 30 percentile of genes in each cell. **Figure S13.** Monoallelic expression in subsampled neurons. **Figure S14.** Numbers of individual cells in which a MA gene was detected. **Figure S15.** Comparison of monoallelic expression between neurons and astrocytes in adult37, adult47 and adult50. (PDF 2190 kb)

Additional file 2: Tables S1, S4 and S5. **Table S1.** Cell numbers used for scRNA-seq of the brains. This table is based on the cell classification in the original study (Darmanis et al., 2015). The column of “Experiment_sample_name” lists the sample labels in the original research. Only the first six adult samples were used in our analysis. **Table S4.** List of disease-related genes showing monoallelic expression in human brains at the cell-type level. **Table S5.** List of module genes from WGCNA. Gene symbols of three significant modules (salmon2, salmon4 and magenta) were listed. (DOC 68 kb)

Additional file 3: Table S2. Gene biased status in each cell of individual brains. The three numbers of SNPs supporting allele bias (MA/BA/Unknown) and the letter indicating gene bias status (M: MA; B: BA; U: Unknown) were separated by slash (/). A dot (.) means data not available. (TXT 5965 kb)

Additional file 4: Table S3. Lists of monoallelic genes in individual cell types. The number of cells supporting the monoallelic gene expression was in column SupportingCellNum and the corresponding single-cell RNA-seq files (GEO accession IDs) were in the column scRNAseqFiles. (XLSX 143 kb)

## Abbreviations

BA: bi-allelic expression
ESC: embryonic stem cell
FPKM: fragments per kilobase of transcript per million mapped reads
hetSNPs: heterozygous SNPs
iPSCs: induced pluripotent stem cells
MA: monoallelic expression
NPC: neuron progenitor cell
OPC: oligodendrocyte progenitor cells
scRNA-seq: single-cell RNA-seq
WGCNA: weighted gene co-expression network analysis
