# Supplementary material for: Characteristics of allelic gene expression in human brain cells from single-cell RNA-seq data analysis
Source: BMC Genomics. 2017 Nov 10;18:860. doi: 10.1186/s12864-017-4261-x (PMC5681780; doi:10.1186/s12864-017-4261-x)
Supplement: Supplementary file 1 — Figure S1. SNP calling result using mouse embryonic scRNA-seq data. Figure S2. A cartoon illustrating the steps and criteria in our allelic expression. Figure S3. Numbers of hetSNP called for the six human brains. Figure S4. The effect of cell numbers on hetSNP calling and the genomic distribution of hetSNPs. Figure S5. Boxplots showing the numbers of brain cells expressing reference (R) or alternative (A) alleles (allelic read depth ≥ 2). Figure S6. Boxplots showing the percentages of reference reads (vs total reads) at hetSNP sites in brain cells (read depth for each of the alleles was ≥2 and the sum of read depths was ≥10). Figure S7. Allelic expression of hetSNPs within human imprinted genes in brain cells. Figure S8. Allelic expression of hetSNPs within mouse imprinted genes in embryonic cells. Figure S9. Numbers of hetSNPs sites with different reference allele ratios. Figure S10. Numbers of hetSNPs sites with different reference allele ratios, after scRNA-seq reads from cells of the same type in individual brains were pooled. Figure S11. Statistical summaries of allelic expression at the gene level. Figure S12. FPKM cutoff values for defining the top 30 percentile of genes in each cell. Figure S13. Monoallelic expression in subsampled neurons. Figure S14. Numbers of individual cells in which a MA gene was detected. Figure S15. Comparison of monoallelic expression between neurons and astrocytes in adult37, adult47 and adult50. (PDF 2190 kb) [file 12864_2017_4261_MOESM1_ESM.pdf]

Zhao et al. supplement

Supplementary Figures S1-S15

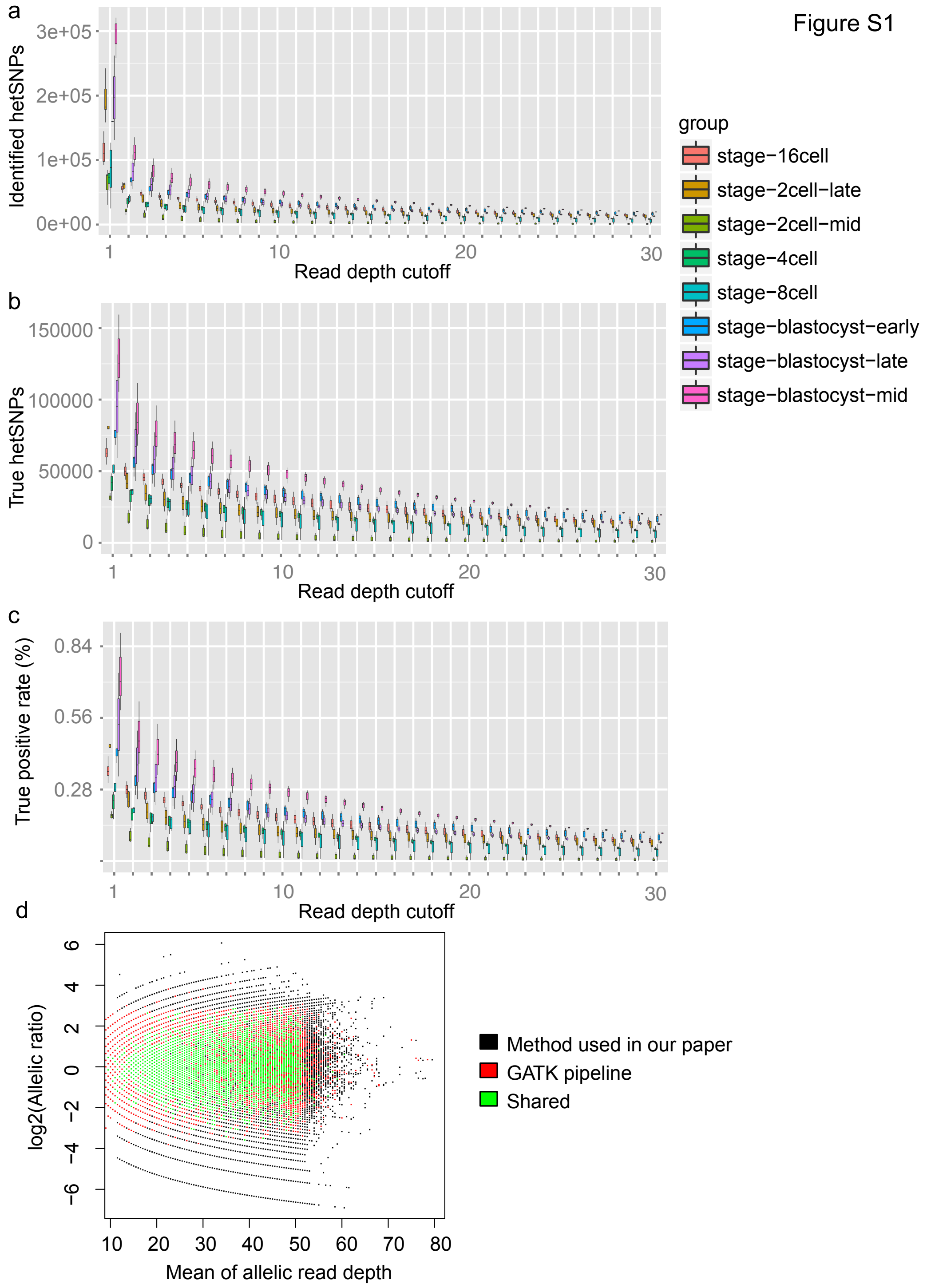

Figure S1. SNP calling result using mouse embryonic scRNA-seq data. Boxplots of hetSNP numbers called at different read depth cutoffs from mouse embryonic scRNA-seq data (a) and the numbers of true hetSNPs compared with the genotyping data (b). (c) True positive rates of SNP calling compared to the full list of hetSNPs existing between the two mouse strains used. (d) Comparison of current SNP calling results with those from the GATK pipeline. Embryos at each stage were processed separately. Cell numbers and read depth were the same as in Fig. 1.

Figure S2

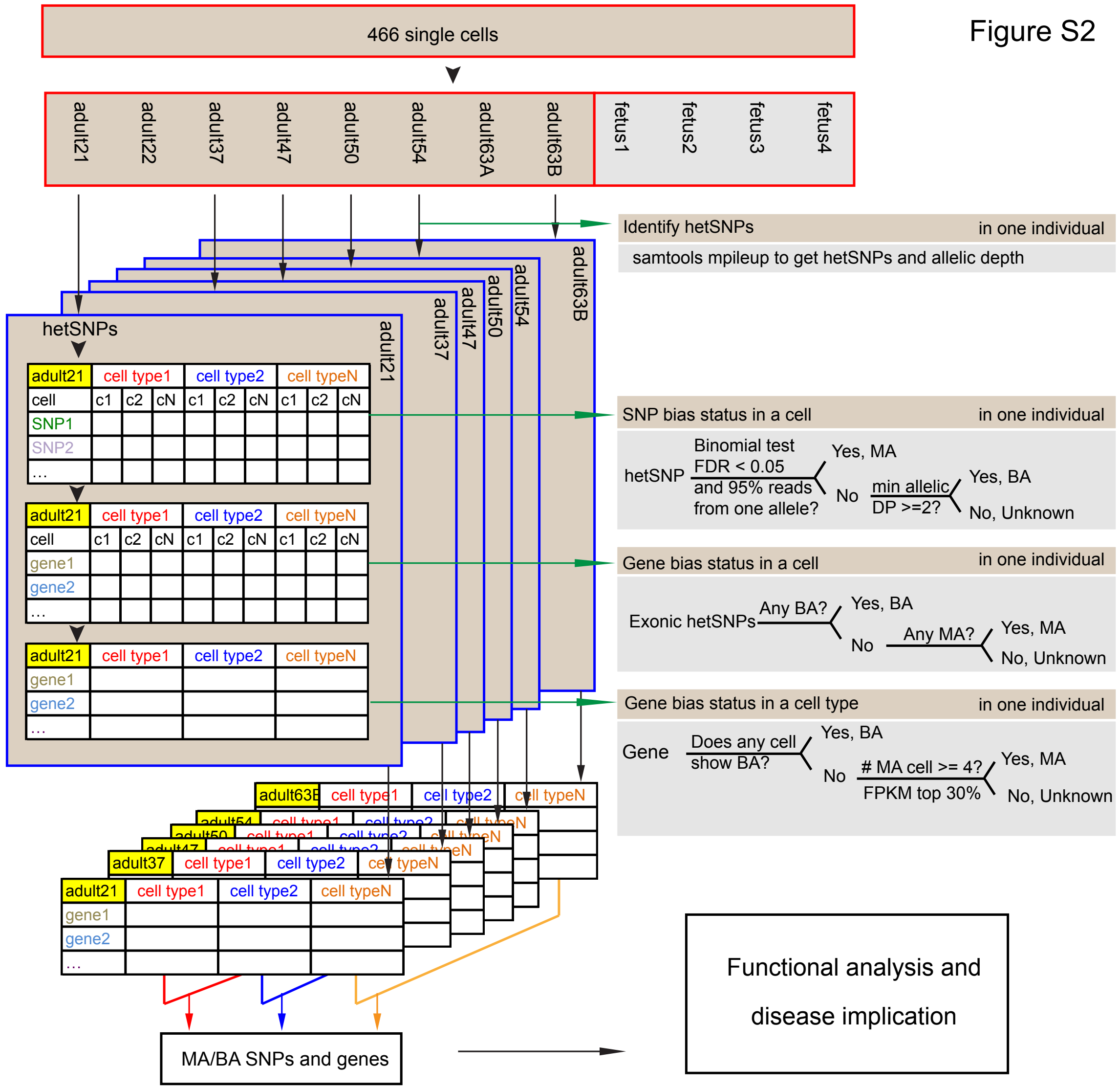

Figure S2. A cartoon illustrating the steps and criteria in our allelic expression. The hetSNPs were called by pooled scRNA-seq data from the same brain. Allelic expression was examined first at the SNP level in each cell, then at the gene level in each cell, and finally at the cell type level in each individual. Functional analysis and co-expression analysis was done on the monoallelically expressed genes at the cell type level.

Figure S3

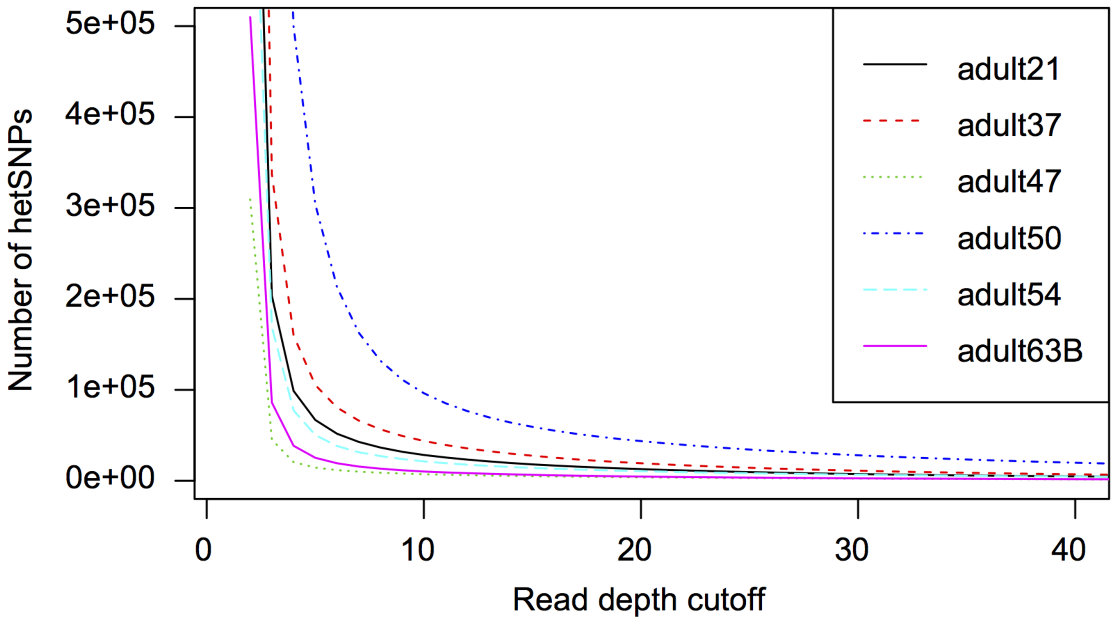

Figure S3. Numbers of hetSNP called for the six human brains. Cells from the same brain were pooled and SNPs were called at a series of read depth cutoff.

Figure S4

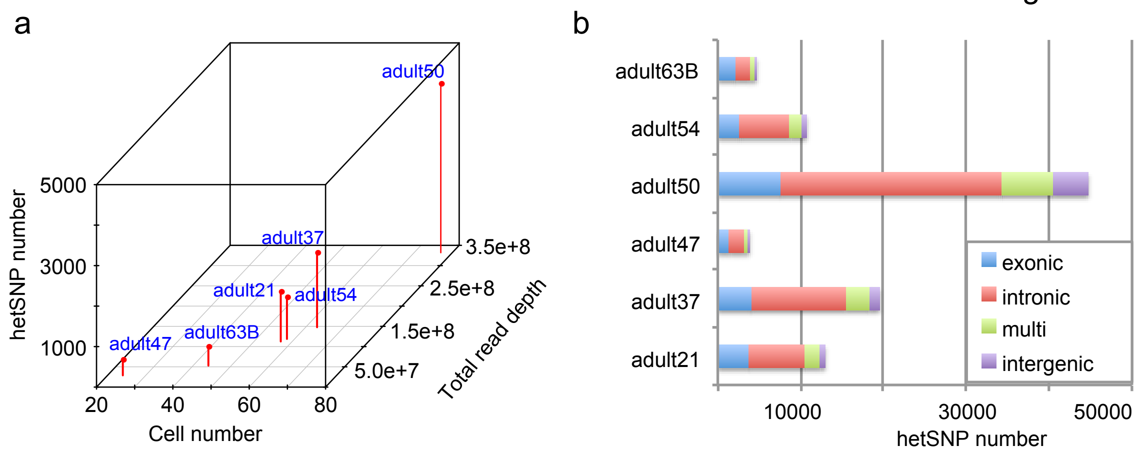

Figure S4. The effect of cell numbers on hetSNP calling and the genomic distribution of hetSNPs. (a). Relationship between hetSNPs called and cell numbers in six adult brains. (b). Genomic distribution of hetSNPs based on Ensembl genome annotation (release 74).

Figure S5

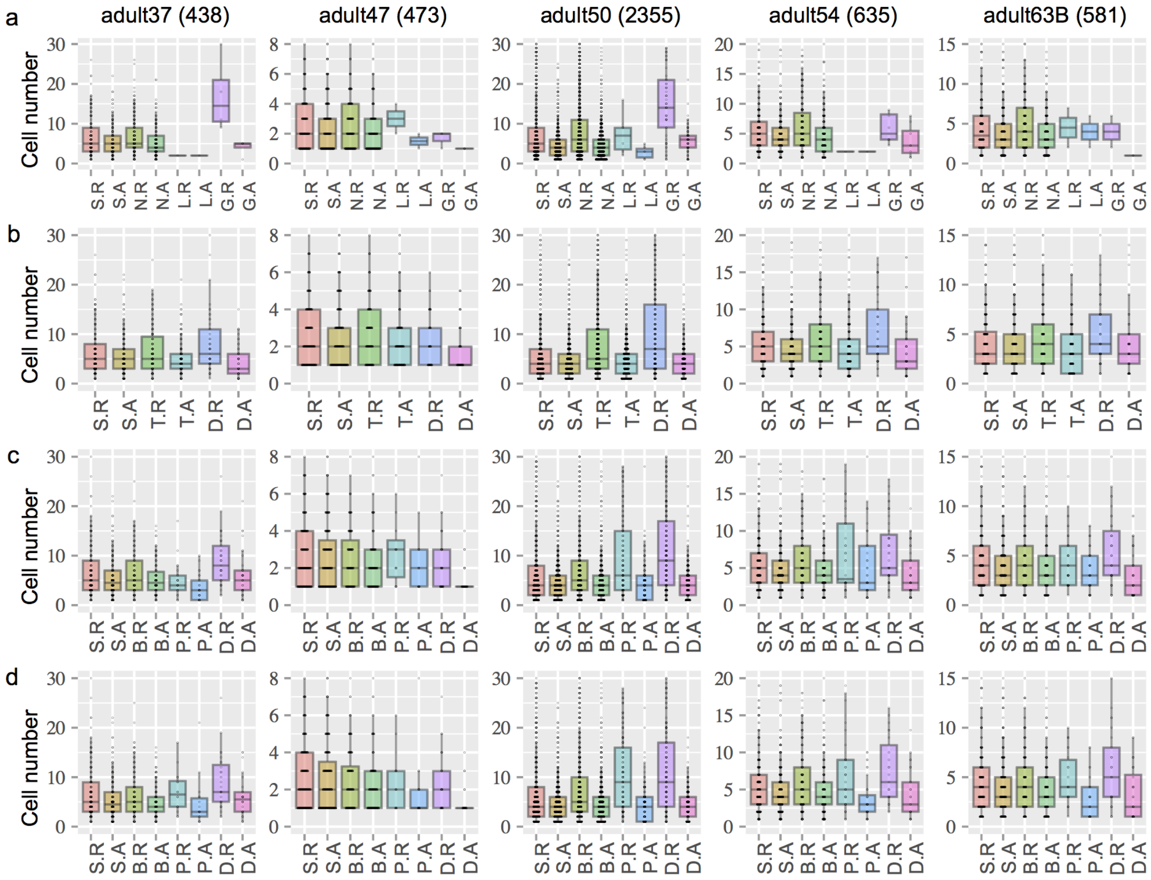

Figure S5. Boxplots showing the numbers of brain cells expressing reference (R) or alternative (A) alleles (allelic read depth ≥ 2). Each point represents a heterozygous SNP whose classification was derived from the exome summary results by wANNOVAR annotation (see Methods). See Fig. 2 legends for SNP classification. (a) SNV: S (n = 203,252,940,323,299) (for adult37, adult47, adult50, adult54, and adult63B respectively; the same below), N (n = 224,215,1307,301,275), L (n = 1,2,6,1,2), G (n = 4,3,89,8,2). (b) SIFT: S (n = 223,258,841,337,308), T (n = 157,150,908,235,206), D (n = 52,57,399,53,57). (c) Polyphen2_HDIV: S (n = 228,267,916,340,314), B (n = 138,145,685,200,171), P (n = 28,15,186,40,42), D (n = 38,38,361,45,44). (d) Polyphen2_HVAR: S (n = 228,267,916,340,314), B (n = 158,154,799,224,197), P (n = 18,16,169,36,27), D (n = 28,28,264,25,33).

Figure S6

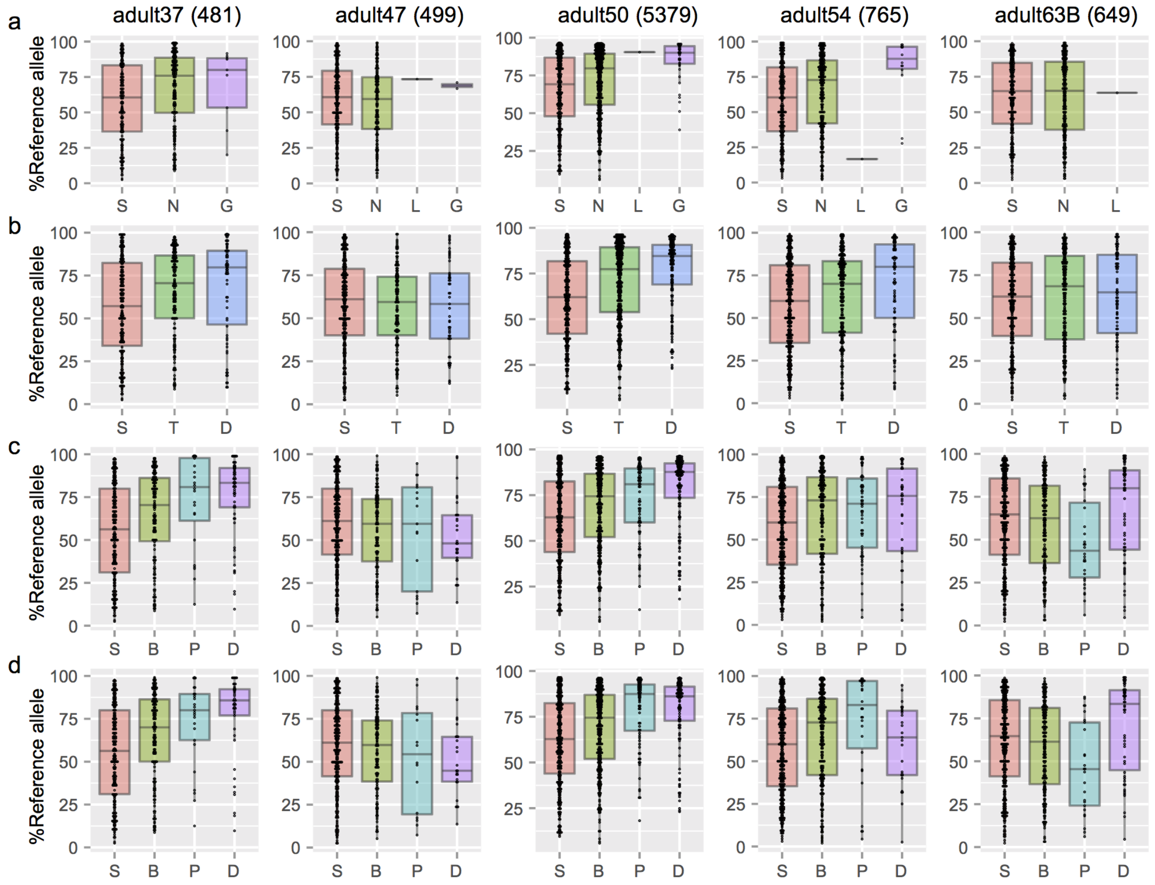

Figure S6. Boxplots showing the percentages of reference reads (vs total reads) at hetSNP sites in brain cells (read depth for each of the alleles was ≥2 and the sum of read depths was ≥10). See Fig. 2 legends for SNP classification.

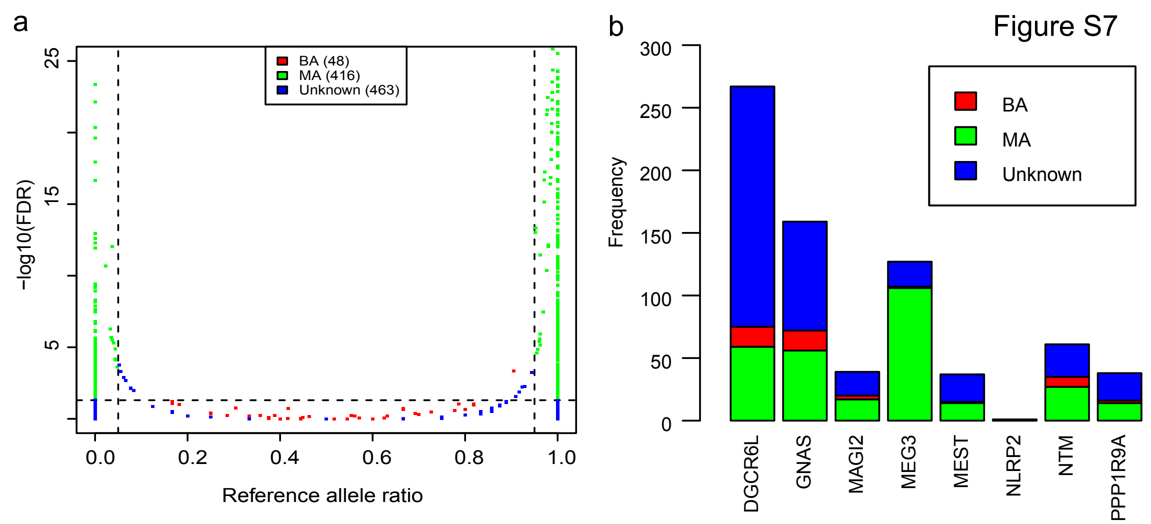

Figure S7. Allelic expression of hetSNPs within human imprinted genes in brain cells. (a) Allelic expression of hetSNPs within imprinted genes in individual cells. Forty-eight instances of hetSNPs were called for BA expression, 416 for MA expression, and 463 for Unknown. The 48 BA instances were from 23 unique hetSNP sites in 8 genes (DGCR6L, GNAS, MAGI2, MEG3, MEST, NLRP2, NTM and PPP1R9A). (b) The allelic expression of all hetSNPs in the 8 genes with BA alleles.

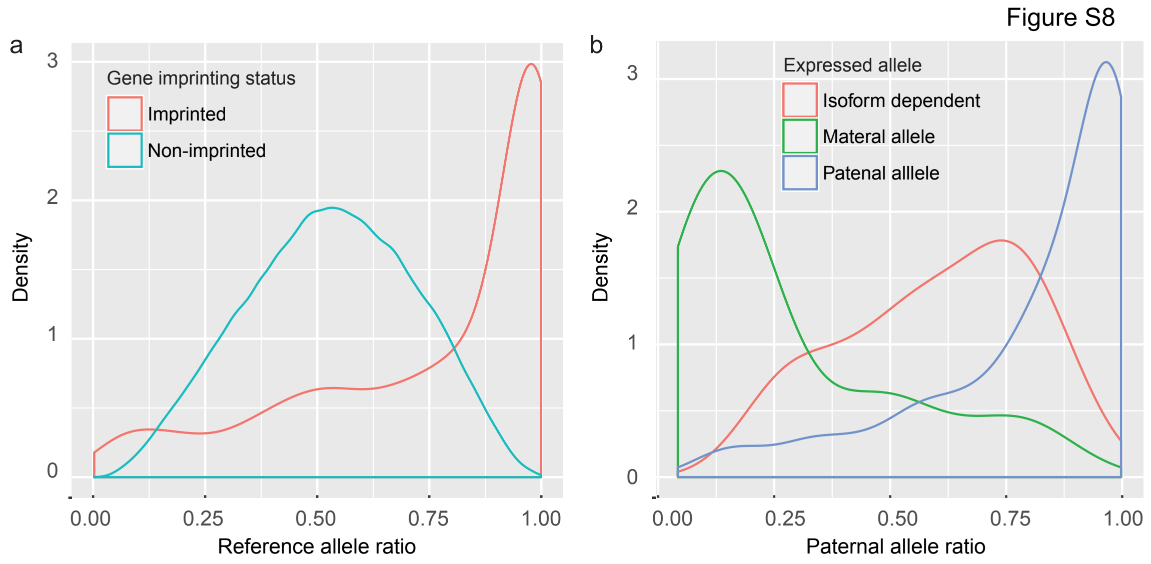

Figure S8. Allelic expression of hetSNPs within mouse imprinted genes in embryonic cells. (a) Distribution of reference allele ratios for imprinted and non-imprinted genes. (b) Distribution of paternal allele ratios for imprinted genes grouped by their expressed alleles, indicating reference bias in a) is likely related to mouse strains used for cross, as one of the parental strains for the scRNA-seq data is C57BL/6, the mouse strain used to generate the reference genome.

Figure S9

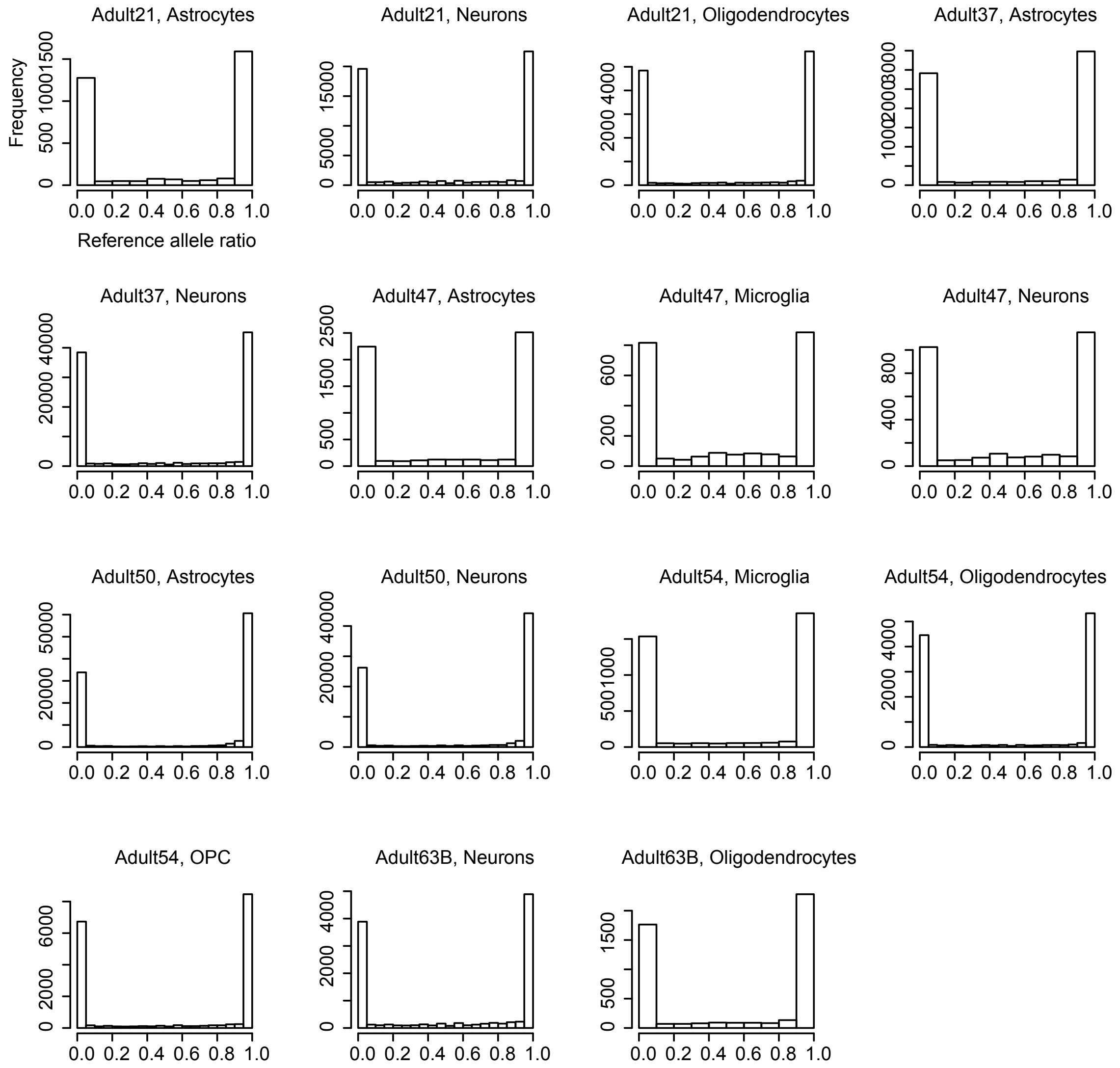

Figure S9. Numbers of hetSNPs sites with different reference allele ratios. Reference allele ratios were calculated for individual hetSNP sites using scRNA-seq reads in each cell, but the histogram shows the distributions after data from cells of the same types and individuals were grouped.

Figure S10

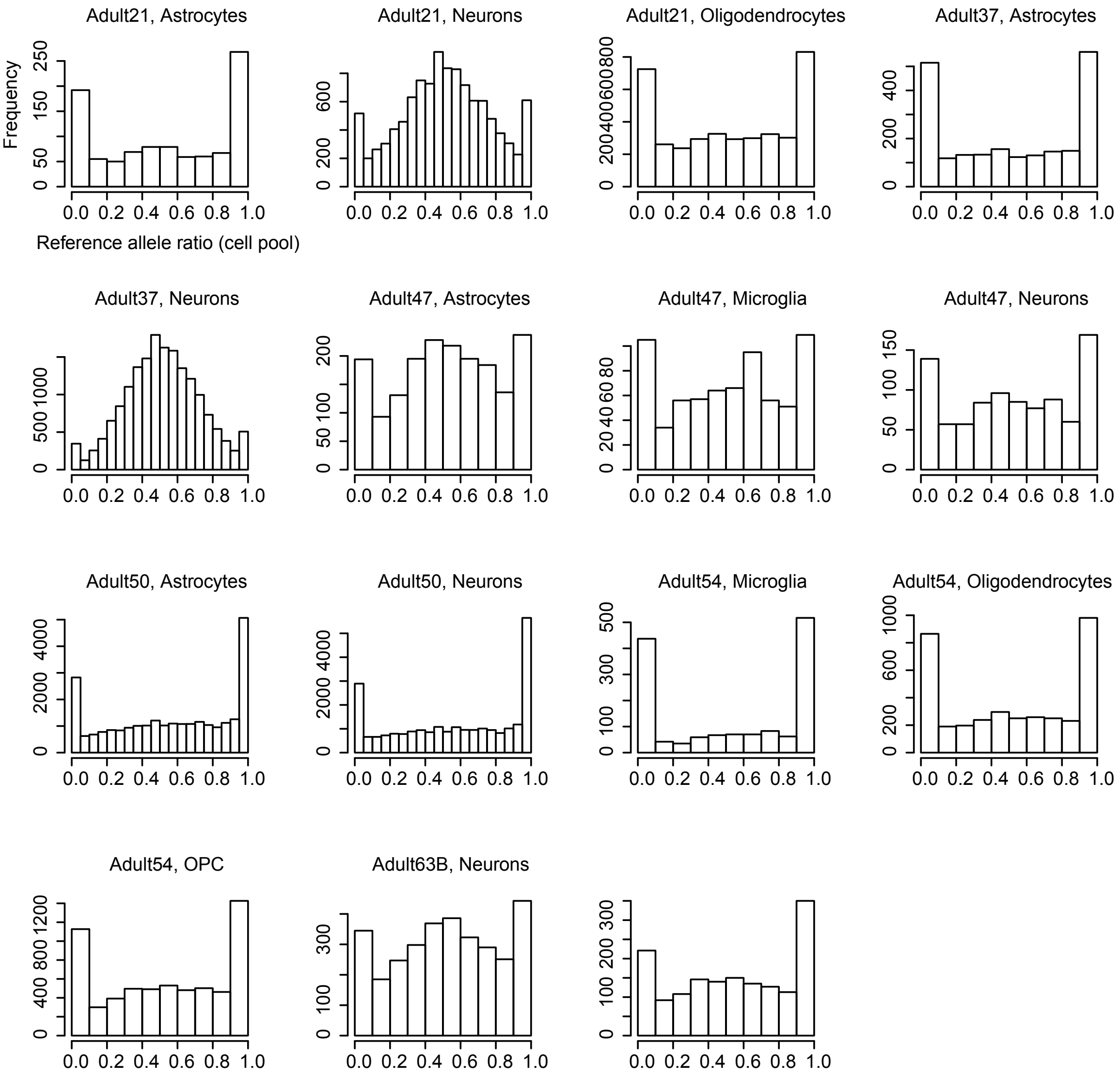

Figure S10. Numbers of hetSNPs sites with different reference allele ratios, after scRNA-seq reads from cells of the same type in individual brains were pooled.

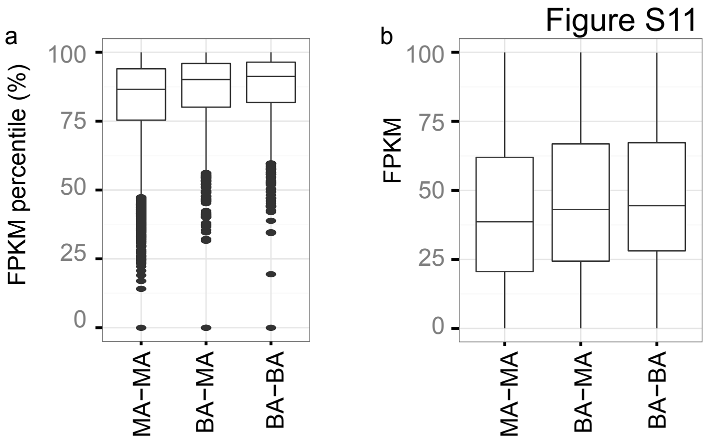

Figure S11. Statistical summaries of allelic expression at the gene level. (a) and (b) Expression levels of genes with two exonic hetSNPs.

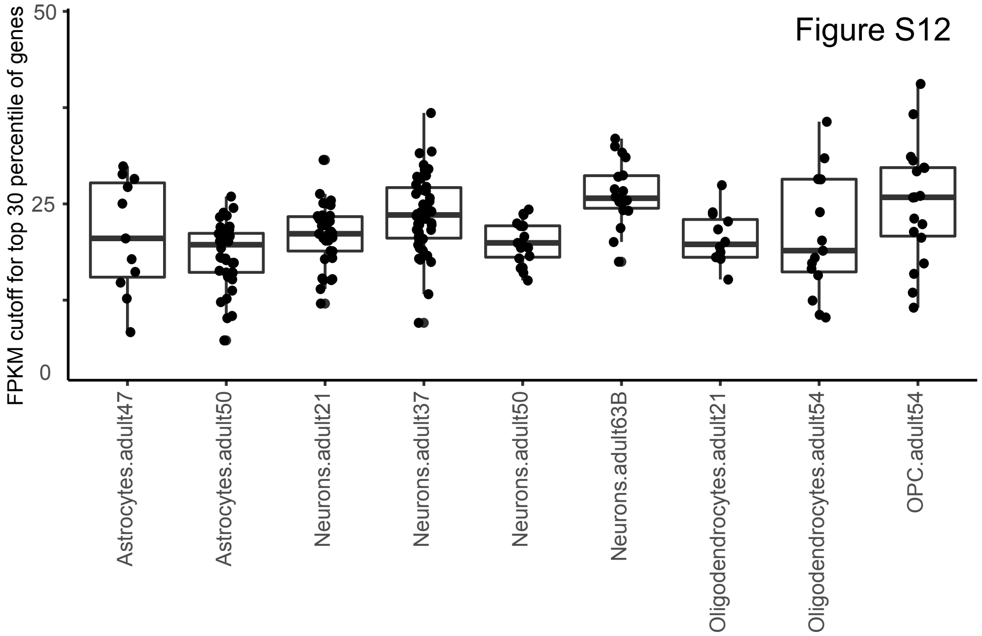

Figure S12. FPKM cutoff values for defining the top 30 percentile of genes in each cell. Each dot represents one cell. Cell types with less than 10 cells in an individual were not shown.

Figure S13

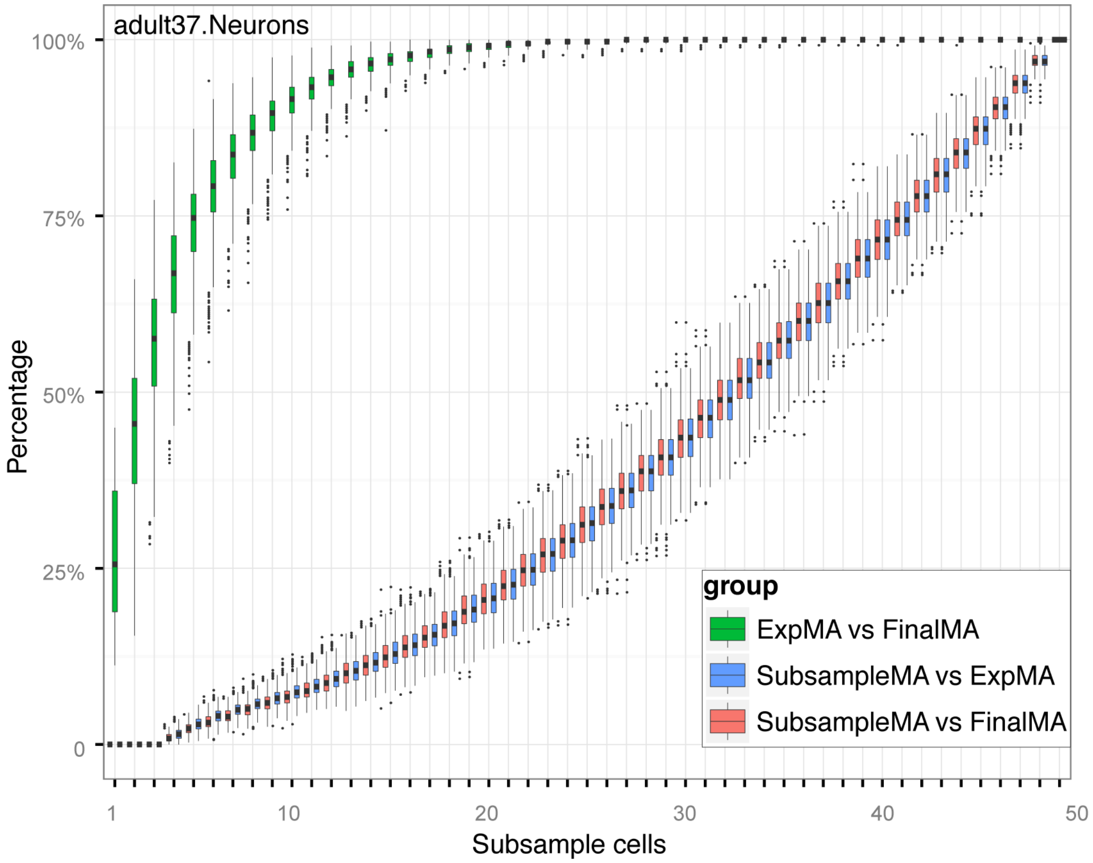

Figure S13. Monoallelic expression in subsampled neurons. Adult37 neurons were randomly subsampled for 1000 times (or use all combinations if all possible combinations are less than 1000) to obtain the MA genes. The MA list in each subsample (SubsampleMA) was compared against the expressed MA list (ExpMA) or final MA list (FinalMA, called with all 50 neurons) to compute the overlapping percentages (y-axis).

Figure S14

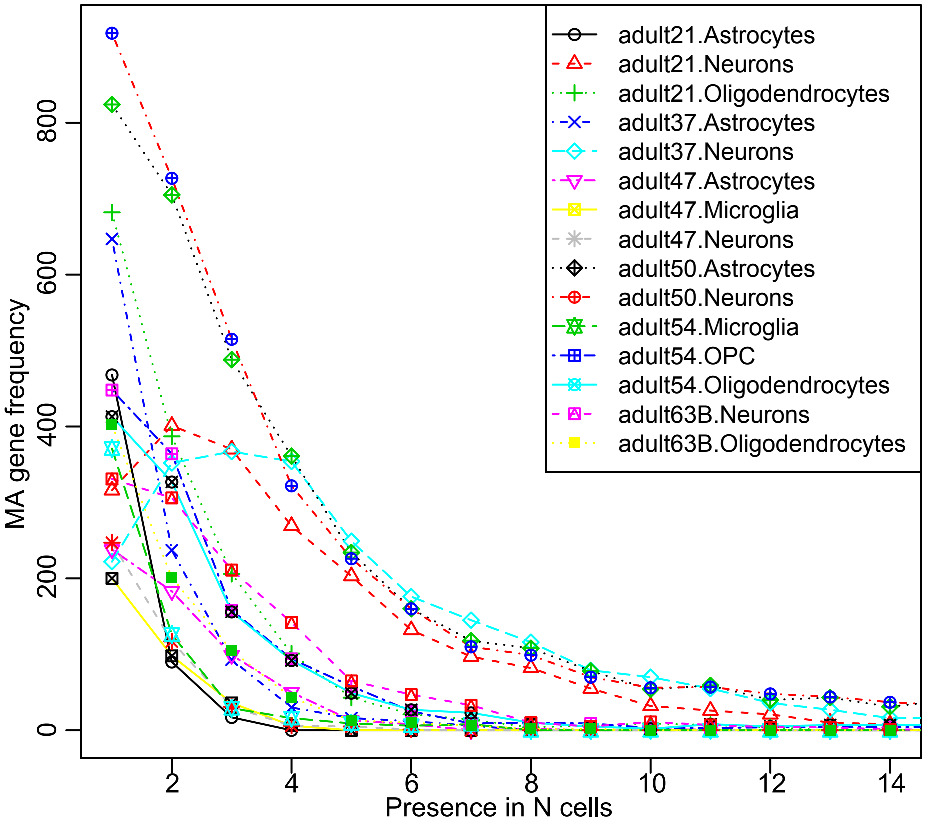

Figure S14. Numbers of individual cells in which a MA gene was detected. MA genes were first identified in each cell and then the cell numbers a gene called MA was counted. The resulting data is plotted as a frequency distribution of MA genes in cells.

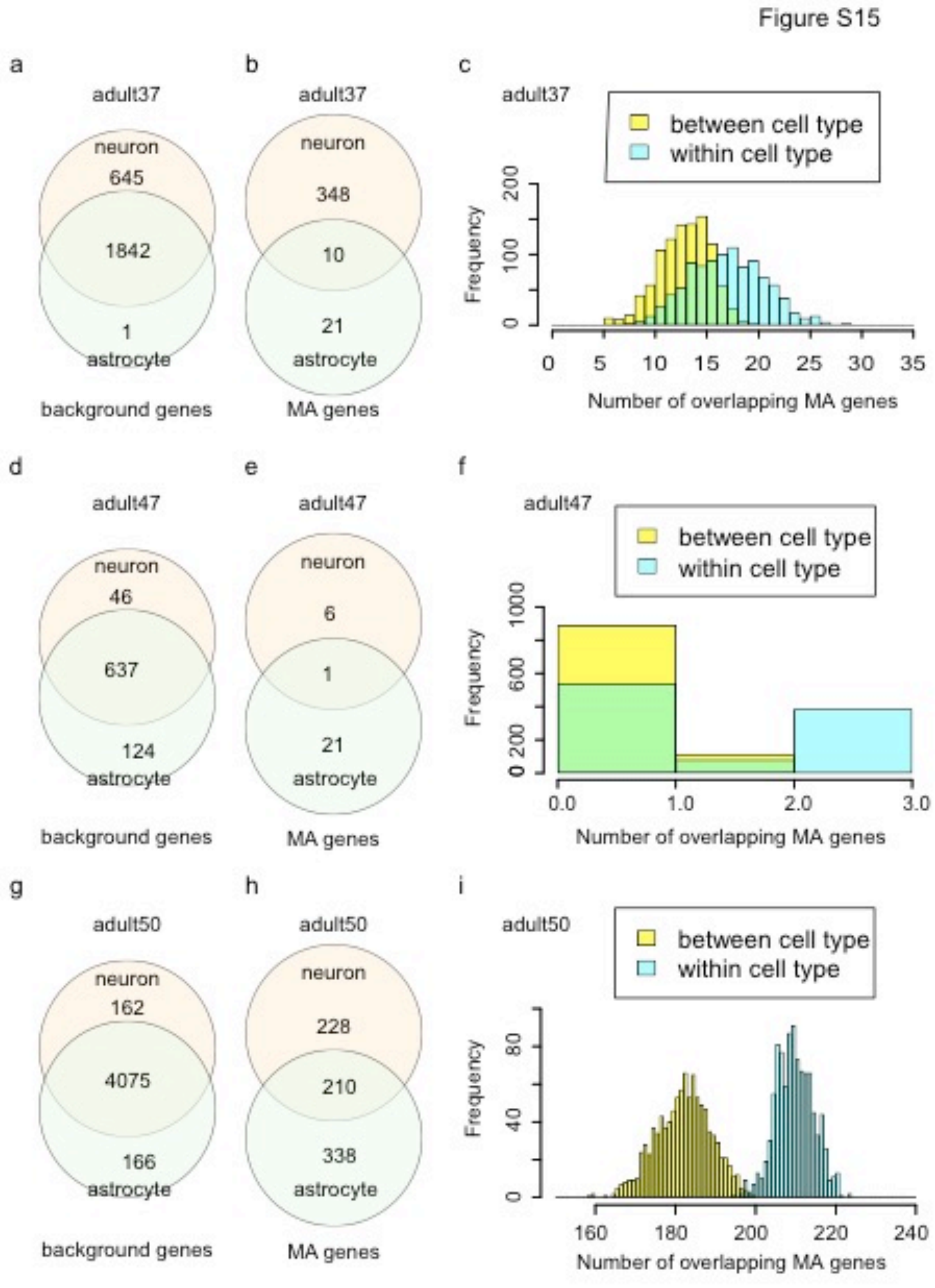

Figure S15. Comparison of monoallelic expression between neurons and astrocytes in adult37, adult47 and adult50. Venn diagrams (a,d,g) show the expressed genes with hetSNPs (at least one allelic read) in the two cell types. Venn diagrams (b,e,h) show the MA genes in the two cell types. Histograms (c,f,i) show the overlap from comparisons of randomly sampled cells (t-test, $p < 2.2e-16$) (See Methods).
