# Supplementary material for: Characteristics of allelic gene expression in human brain cells from single-cell RNA-seq data analysis
Source: BMC Genomics. 2017 Nov 10;18:860. doi: 10.1186/s12864-017-4261-x (PMC5681780; doi:10.1186/s12864-017-4261-x)
Supplement: Supplementary file 2 — Table S1. Cell numbers used for scRNA-seq of the brains. This table is based on the cell classification in the original study (Darmanis et al., 2015). The column of “Experiment_sample_name” lists the sample labels in the original research. Only the first six adult samples were used in our analysis. Table S4. List of disease-related genes showing monoallelic expression in human brains at the cell-type level. Table S5. List of module genes from WGCNA. Gene symbols of three significant modules (salmon2, salmon4 and magenta) were listed. (DOC 68 kb) [file 12864_2017_4261_MOESM2_ESM.doc]

**Additional file 2:Supplementary tables S1, S4 and S5**

**Table S1.** **Cell numbers used for scRNA-seq of the brains.** This table is based on the cell classification in the original study (Darmanis et. al., 2015). The column of “Experiment_sample_name” lists the sample labels in the original research. Only the first six adult samples were used in our analysis.

|  | **Experiment_sample_name** | **Astrocytes** | **Endothelial** | **Microglia** | **Neurons** | **Oligodendrocytes** | **OPC** | **Fetal_quiescent** | **Fetal_replicating** | **hybrid** | **hybrids** | **sum** |
| --- | --- | --- | --- | --- | --- | --- | --- | --- | --- | --- | --- | --- |
| adult21 | AB_S7 | 3 | 0 | 0 | 35 | 12 | 0 | 0 | 0 | 7 | 0 | 57 |
| adult37 | AB_S11 | 7 | 1 | 1 | 50 | 1 | 0 | 0 | 0 | 0 | 3 | 63 |
| adult47 | AB_S1 | 11 | 2 | 5 | 6 | 0 | 0 | 0 | 0 | 0 | 0 | 24 |
| adult50 | AB_S4 | 38 | 0 | 0 | 19 | 1 | 0 | 0 | 0 | 0 | 19 | 77 |
| adult54 | AB_S8 | 1 | 0 | 7 | 0 | 15 | 18 | 0 | 0 | 17 | 0 | 58 |
| adult63B | AB_S5 | 0 | 16 | 1 | 18 | 9 | 0 | 0 | 0 | 0 | 0 | 44 |
| adult22 | AB_S3 | 0 | 1 | 0 | 2 | 0 | 0 | 1 | 0 | 0 | 0 | 4 |
| adult63A | AB_S2 | 2 | 0 | 2 | 1 | 0 | 0 | 0 | 0 | 0 | 0 | 5 |
| fetus1 | FB_S1 | 0 | 0 | 0 | 0 | 0 | 0 | 5 | 21 | 0 | 0 | 26 |
| fetus2 | FB_S2 | 0 | 0 | 0 | 0 | 0 | 0 | 44 | 2 | 0 | 0 | 46 |
| fetus3 | FB_S3 | 0 | 0 | 0 | 0 | 0 | 0 | 33 | 0 | 0 | 0 | 33 |
| fetus4 | FB_S4 | 0 | 0 | 0 | 0 | 0 | 0 | 27 | 2 | 0 | 0 | 29 |
| sum |  | 62 | 20 | 16 | 131 | 38 | 18 | 110 | 25 | 24 | 22 | 466 |

**Table S4. List of disease-related genes showing monoallelic expression in human brains at the cell-type level**.

| **Group** | **Cell-type MA genes** |
| --- | --- |
| Haploinsufficiency | TCF4, RPS4X, SRGAP3, FBXW7, XRCC5, ID2, ATP1A2, FGFR3, BUB3, ANK2, PRKAR1A, TFRC, NF1, ADAR, PROX1, PMP22, EGR1, APC, YWHAE, CAMTA1 |
| Autism spectrum disorder | ABAT, ADNP, ANK2, ARNT2, ASB8, ATCAY, ATRX, BIRC6, BRWD1, CACNA1E, CASD1, CDH10, CHD2, CNTN4, CNTNAP2, DLX6, DST, EIF4E, GABRB3, GGNBP2, GLO1, GRIA3, GRIK2, GRIP1, KCND2, KHDRBS2, LEO1, LRFN5, NDUFB5, NF1, NRCAM, NRXN3, PAFAH1B1, PCDH9, RAB2A, RNF38, RPL10, SCN2A, SETD2, SETD5, SHANK2, SLC1A1, SPARCL1, TBL1XR1, TUBA1A, TUBGCP4, UBR3, UNC80, VIP, YWHAE |
| Schizophrenia | ABAT, ACP1, ANKRD12, APC, APP, AQP4, BLOC1S2, CALM1, CAPZA2, CIT, CNP, CNTN1, CPLX1, DPYSL2, EGR1, ENO1, ERBB4, ESD, FGF14, FKBP1A, GABBR1, GABRA2, GAP43, GLS, GLUL, GNAL, GNAZ, GNPAT, GPR158, GRIA2, GRIA3, GRIA4, GRIK2, GRIP1, HK1, HOMER1, HSPA5, HTR5A, LGI1, MAGI3, MAP1A, NCOA7, NQO2, NRXN3, NTRK2, PAFAH1B1, PER2, PGBD1, PLCE1, PLP1, PPP3CA, PRNP, SAT1, SLC1A1, SLC1A2, SLC1A3, SLC25A27, SMARCA2, SPARCL1, SPTAN1, TCF4, TIMP3, TUBA1A, UHMK1, YWHAB, YWHAE, ZNF804A |

**Table S5. List of module genes from WGCNA**. Gene symbols of three significant modules, or salmon2, salmon4 and magenta, were listed.

| **Module** | **Genes** |
| --- | --- |
| salmon2 | MOBP, ELOVL1, CLDN11, MAPK8IP1, SLAIN1, PIP4K2A, PTMA, RNASE1, ERMN, SLC12A2, MYRF, SEPT4, SCD, CAPN3, FEZ1, TMEM144, TF, PLP1, ENPP2, FA2H, PLLP, CNTN2, PPP1R14A, ZDHHC9, QDPR, DBNDD2, CLDND1, HSPA2, MOG, TUBB4A, OPALIN, KLK6, CARNS1, MBP |
| salmon4 | PPAP2B, GABRA2, EDNRB, DST, GJB6, DIO2, ALDOC, SLC7A11, GJA1, GPR37L1, EZR, ETNPPL, GLUL, AGT, SLC1A2, F3, SPARCL1, MGST1, CPE, CLDN10, SOX9, PON2, DDAH1, AQP4, ATP1A2, SLCO1C1, FAM171B, SDC4, MT3, LRRC16A, FGFR3, IL33, ATP1B2, RANBP3L, AHCYL1, RYR3, NTRK2, ENO1, CST3, CKB, CLU, GPR98, TPD52L1, BMPR1B, PLTP, ITM2C, SFXN5, MT1E, PREX2, GLUD1, SLC1A3, C1orf61 |
| magenta | RPL19, SV2A, SVOP, FXYD6, NGFRAP1, GPX4, GAS7, ATP6V1F, SEZ6L2, KCNJ3, SNCA, MDH1, CHGB, PNMA2, ATP6V1G2, MAPT, SNAP25, TSPYL1, EIF4A2, CNTNAP2, GRIN2B, TBC1D24, NSG2, ELAVL4, CBX6, NRIP3, UCHL1, GOT2, GAD2, BEX4, GOT1, PGM2L1, ATP6V1A, SLC4A10, NDRG4, SYNPR, BEX2, SYN1, REEP1, GUCY1B3, NSG1, SLC25A4, SOD1, NAP1L5, PAK3, RTN1, MAGEE1, GABRB2, COX6C, MAP1B, YWHAH, SCN2A, GABRA1, ADD2, CAP2, SCG5, NECAP1, MYT1L, YWHAG, TCEAL3, HINT1, AC013449.1, GAD1, MAP2, SPRYD3, SCG2, GAP43, GNG3, SYT1, NAP1L3, MLLT11, TUBA4A, NAPB, CALM1, RAB3A, PARM1, VSTM2A, USP11, PIN1, OAZ1, GRIK2, FGF14, VSNL1, SCN3B, RTN3, STXBP1, COX7C, CALB2, PREPL, RAB3C, FRRS1L, ROBO2, AP1S2, ATCAY, PAIP2, PLK2, OXCT1, SLC22A17, CELF4, THY1, STMN2, TMEM14A, DYNLL1, CCK, PNMAL1, BEX5, COX4I1, CAMK2A, NEDD4L, GABBR2, HPRT1, PEBP1, TSPAN7, NCDN, HSP90AB1, PEG3, USMG5, BEX1, ARL4C, NDUFC2, SARS, GRIA1, NDUFA4, ENC1, GPI, GABRG2, SYNGR3, ALDOA, NDUFA1, NRXN3, FGF12, PTPRN, STMN1, SERINC1, TSPYL2, TMEM130, ATPIF1, MAP7D2, ATP1B1, NRSN2, REEP5, KIF5C, ENO2, RBFOX2, RIMS2, CHRM3, SERPINI1 |
